# Attenuated Infectious Bronchitis Virus (IBV) Vaccination Induces Protective Humoral and Cellular Immunity Against SARS‐CoV‐2 in Mammals

**DOI:** 10.1155/jimr/9309800

**Published:** 2026-03-29

**Authors:** Ney de Carvalho Almeida, Mauricio Fraga van Tilburg, Bruno Bezerra da Silva, João Xavier da Silva Neto, Luiz Francisco Wemmenson Gonçalves Moura, Natália do Vale Canabrava, Diane Isabelle Magno Cavalcante, Deysi Viviana Tenazoa Wong, Roberto César Pereira Lima, Daniel Pascoalino Pinheiro, Dayane Alves Costa, Danúbio Andrade Bezerra Farias, Pablo Abreu de Morais, Valder Nogueira Freire, Valdir Ferreira de Paula, Francisco Franciné Maia, Diego Veras Wilke, Gilvan Pessoa Furtado, Eridan Orlando Pereira Tramontina Florean, Maria Izabel Florindo Guedes

**Affiliations:** ^1^ Biotechnology and Molecular Biology Laboratory, State University of Ceará, Campus do Itaperi, Fortaleza, 60714-903, Ceará, Brazil, uece.br; ^2^ Department of Pathology and Forensic Medicine, Faculty of Medicine, Federal University of Ceará, Fortaleza, Brazil, ufc.br; ^3^ Laboratory of Inflammation and Cancer Pharmacology, Drug Research and Development Center (NPDM), Department of Physiology and Pharmacology, Federal University of Ceará, Fortaleza, Brazil, ufc.br; ^4^ Oswaldo Cruz Foundation in Ceará, Eusebio, Brazil; ^5^ Federal Institute of Education, Science and Technology of Ceará, Campus Horizonte, Horizonte, Ceará, Brazil; ^6^ Department of Physics, Federal University of Ceará, Fortaleza, Ceará, Brazil, ufc.br; ^7^ Department of Natural Sciences, Mathematics and Statistics, Universidade Federal Rural do Semi-Árido, Mossoró, 59625-900, Rio Grande do Norte, Brazil, ufersa.edu.br; ^8^ Physiology and Pharmacology Department, Federal University of Ceara, Fortaleza, Ceará, Brazil, ufc.br

**Keywords:** alternative vaccine, cross-protection, infectious bronchitis virus, respiratory infection, vaccine protection

## Abstract

Occupational exposure to infectious bronchitis virus (IBV) has been associated with cross‐reactivity to other coronaviruses such as SARS‐CoV‐2. In this study, we investigated the immune response of IBV using reconstituted samples of the commercial H120 Mass‐I vaccine (Zoetis) against SARS‐CoV‐2 infection. Bioinformatics analyses, serological assays, and neutralization tests were conducted to evaluate the neutralizing capacity of anti‐IBV antibodies against SARS‐CoV‐2 in serum samples from poultry farm workers exposed to the IBV‐H120 vaccine. Mouse immunization assays and flow cytometry analysis were conducted to characterize the immune response triggered by IBV‐H120. Finally, a histological analysis of the lungs of Syrian hamsters vaccinated with IBV and challenged with SARS‐CoV‐2 (Wuhan and Delta strains) was conducted to evaluate the protective potential of the vaccine. Neutralizing antibodies against IBV‐H120 were detected in the serum of poultry farm workers, indicating cross‐reactivity with SARS‐CoV‐2. The immune response in mice immunized with IBV‐H120 demonstrated that only the intranasal and oral routes were effective against SARS‐CoV‐2. An increase in the percentage of CD19^+^ induced by IBV‐H120 vaccination was demonstrated by flow cytometry. Intranasal administration of the IBV‐H120 vaccine reduced lung damage in Syrian hamsters against the Wuhan and Delta variants of SARS‐CoV‐2. IBV‐H120 elicited both humoral and cellular immune responses in mice and reduced lung damage in hamsters challenged with the Wuhan and Delta variants. This indicates that it could be an improved vaccine for controlling SARS‐CoV‐2.

## 1. Background

The 2019 coronavirus disease (COVID‐19) is a viral infection caused by the severe acute respiratory syndrome coronavirus 2 (SARS‐CoV‐2). SARS‐CoV‐2 is a virus belonging to the *Betacoronavirus* genus of the Coronaviridae family. Like other members of this family, SARS‐CoV‐2 has an enveloped viral particle containing the viral proteins S (Spike), M (Membrane), and E (Envelope) [1]. COVID‐19 has become a significant global health issue, being declared a Public Health Emergency of International Concern from March 2020 to May 2023, with over 766 million confirmed cases and approximately 6.9 million deaths worldwide [2]. Vaccination is widely regarded as the most effective way to prevent, control, and reduce COVID‐19‐related morbidity and mortality, with several vaccines rapidly developed and licensed for this purpose [3]. However, despite this swift development, global immunization coverage remains below desired levels, especially in low‐income countries [4]. The emergence of SARS‐CoV‐2 variants and other potential pandemic respiratory viruses highlights the need for new strategies to induce stable and long‐lasting immune responses [5].

The idea of using pathogens that cause diseases in animals for the development of vaccines for humans began in the 18th century with the British physician Edward Jenner. He inoculated a child with pus from a pustule caused by the cowpox virus, and 6 weeks later, the child was exposed to the human smallpox virus but did not develop the disease [6]. Vaccination based on the use of the cowpox virus in humans quickly spread throughout Europe and across the world [7].

Besides humans, viruses from the Coronaviridae family infect a wide range of mammals and birds. The first coronavirus identified was the infectious bronchitis virus (IBV), part of the gamma coronavirus genus, which causes an acute and highly contagious respiratory disease in chickens [8]. Vaccines against IBV, especially those prepared with strains from the Massachusetts serotype, offer broad antigenic protection and are commonly included in most poultry vaccination programs [9].

Almeida and Tyrrell [10] reported morphological similarities between the avian IBV and human coronaviruses. Although the symptomatology of IBV in birds resembles that of COVID‐19, there are no reports in the literature of IBV causing disease in humans. Despite belonging to the same family, IBV and human coronaviruses are classified into distinct genera. Avian infectious bronchitis is not classified as a zoonosis by the World Organisation for Animal Health. Workers exposed to this virus in occupational settings do not develop illness. Such exposure may result in the production of antibodies that recognize not only IBV but also conserved regions of proteins shared with other members of the Coronaviridae family, including SARS‐CoV‐2 [11]. In 1968, researchers at the University of Rhode Island detected neutralizing antibodies against IBV in serum from poultry workers, suggesting exposure without evidence of human infection. Subsequent studies confirmed similar findings in research conducted in the Northeastern United States [12, 13].

Accordingly, the present study provides the first report of the use of an attenuated IBV vaccine in mammals, demonstrating its protective efficacy against SARS‐CoV‐2 infection in hamsters.

## 2. Results

### 2.1. Evaluation of IBV‐H120 Replication in Mammalian Cells

The results showed unaltered Ct values across all cell lines throughout the analyzed period, indicating an absence of viral replication. These findings reinforce that IBV‐120’s replication capacity in mammalian cells is minimal, further supporting the literature on the virus’s host specificity for avian species.

### 2.2. Serological Testing of Serum Samples From Poultry Farm Workers

The chemiluminescence assay revealed that 50% (5/10) of workers from poultry farms workers exhibited an IgG response to anti‐S1‐SARS‐CoV‐2. When the same samples were analyzed using an indirect enzyme‐linked immunosorbent assay (ELISA) with purified SARS‐CoV‐2 and IBV‐H120 vaccine as antigens, different immunoglobulin isotypes (IgG, IgA, and IgM) reactive to both viruses were detected. The results, presented in Figure S2A,B, show that IgG was detected in 50% (5/10) of samples against SARS‐CoV‐2 and only 20% (2/10) against IBV‐H120; IgA was detected in 90% (9/10) of samples against both SARS‐CoV‐2 and IBV‐H120; IgM was present in 70% (7/10) of samples against SARS‐CoV‐2 and 60% (6/10) against IBV‐H120.

### 2.3. Neutralization Assay of Serum Samples From Poultry Farm Workers

The serum of the individuals (*n* = 10) responsible for the poultry immunization showed a neutralizing capacity against SARS‐CoV‐2 through PRNT. Quantitative analysis revealed a neutralization rate ranging from 53% to 95%, with no reaction observed in the negative control group (*n* = 10; Figure 1A and Figure S2C). However, no direct relationship was found between antibody (anti‐S1‐SARS‐CoV‐2) levels and neutralizing capacity against SARS‐CoV‐2. These results show an independent correlation between serological levels and neutralization (Figure 1B).

Figure 1Viral neutralization test (PRNT) and correlation with detection of IgG antibodies against S1 protein. (A) Viral neutralization test (PRNT) with the serum from poultry farm workers exposed to IBV‐H120 and from a negative control group (*n* = 10, prepandemic). (B) Correlation between viral neutralization levels and levels of IgG antibodies against S1 protein (chemiluminescence). Results are presented as mean ± standard deviation (*M* ± SD). ANOVA and *T*‐student test were performed.  ^∗∗∗∗^ *p* < 0.0001.

**Figure:**
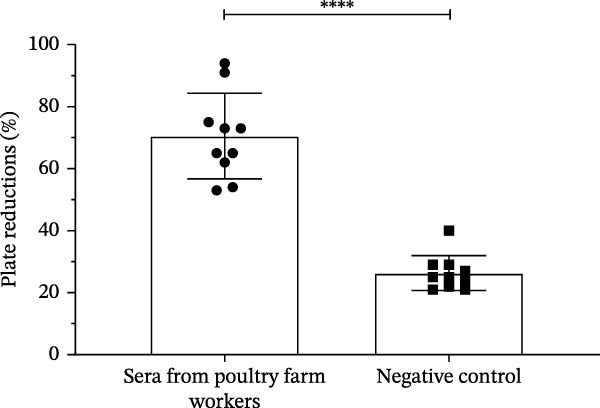
(A)

**Figure:**
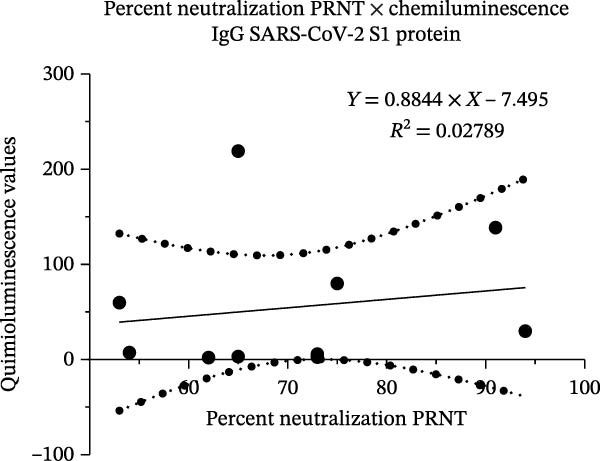
(B)

### 2.4. In Silico Analysis

In the sequencing analysis of IBV and SARS‐CoV‐2, the S domains of the four IBV strains are fully conserved (Figure 2A). However, two mutations are observed in SARS‐CoV‐2 strains at positions 484 and 501 in the receptor‐binding domain (RBD) region. Comparing the viruses, two regions stand out: (i) residues 454–502 show five conserved, seven highly similar, and eight partially similar residues; (ii) residues 503–561 have 13 conserved, eight highly similar, and nine partially similar residues. Figure 2B shows IBV S complexed with an antibody P2B‐2F6, stabilized by HC, and Figure 2C illustrates that the RMSD stabilizes after 80 ns, indicating complex stability.

Figure 2Analysis in silico of IBV S protein and the antibody P2B‐2F6 produced by SARS‐CoV‐2. (A) Sequence alignment of four strains of IBV and four of SARS‐CoV‐2, with red arrows indicating positions 484 and 501 of the SARS‐CoV‐2 RBD. The asterisk “ ^∗^” indicates the conserved residues, the colon “:” indicates replacement with a similar residue, and the dot “.” indicates a low similarity between the residues. (B) Side and top views of the IBV S/P2B‐2F6 antibody complex. The heavy chain of the antibody is represented in red (AbHC), while the light chain (AbLC) is in green, and, finally, the IBV spike protein is in blue. (C) The RMSD of atomic positions with respect to the backbone molecular structure of the complex IBV S/P2B‐2F6 antibody at 300 K obtained from a MD simulation of 120 ns. (D) 2D map of the interaction energies obtained from the MFCC quantum biochemistry description between the AbHC residues (horizontal axis) and the IBV residues (vertical axis). The S1‐NTD domain (blue), the SD1 (magenta), and SD2 (orange) subdomains are highlighted on the vertical axis. The “hot spot” residues are marked in red dashed lines, and their interaction energies (*E*_spot_) are exhibited around the hotspot regions. *E*_marked_ (sum of the *E*_spot’s_) is also displayed, as well as the total energy *E*_TOT_. (E) IBV spike protein in its closed conformation. The sequence of residues 226–256 is highlighted in purple and corresponds to the S1‐NTD domain and the SD2′ and SD1′ parts, while the sequence of residues 490–576 displayed in blue corresponds to the SD1″ and SD2″ parts [14]. (F, G) The binding site, interaction energy, and residue domain (BIRD) panels show the accumulated interaction energies for IBV S protein residues S1‐NTD/SD2′ and SD1″/SD2″, respectively. (H, I) BIRD for the AbHC interacting with the IBV S protein.

**Figure:**
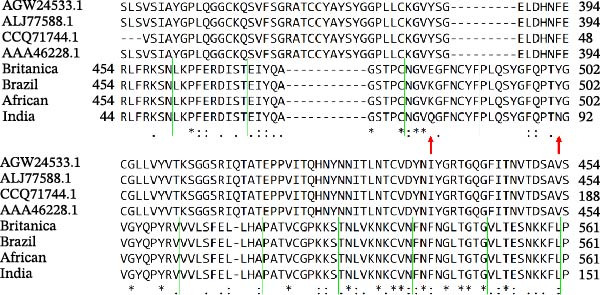
(A)

**Figure:**
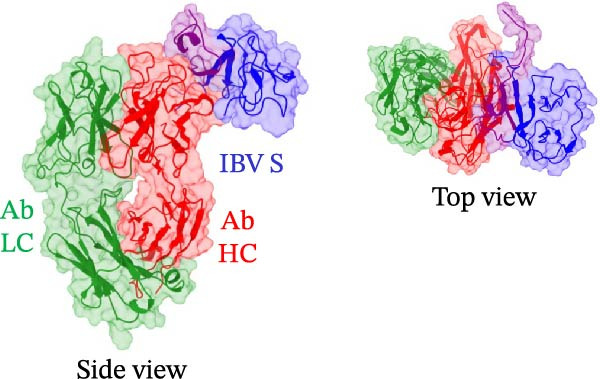
(B)

**Figure:**
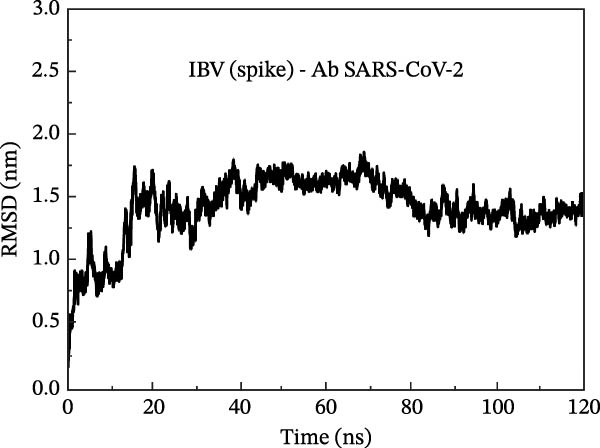
(C)

**Figure:**
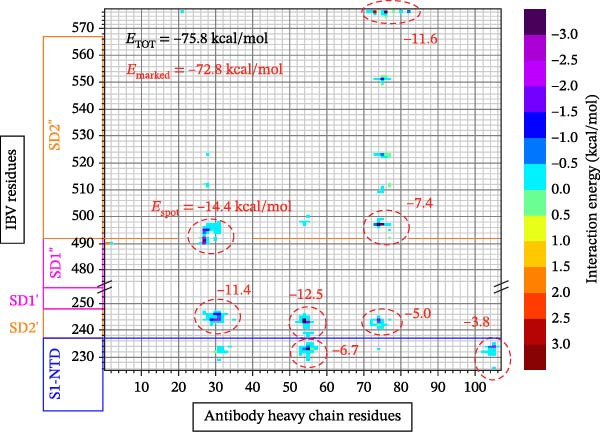
(D)

**Figure:**
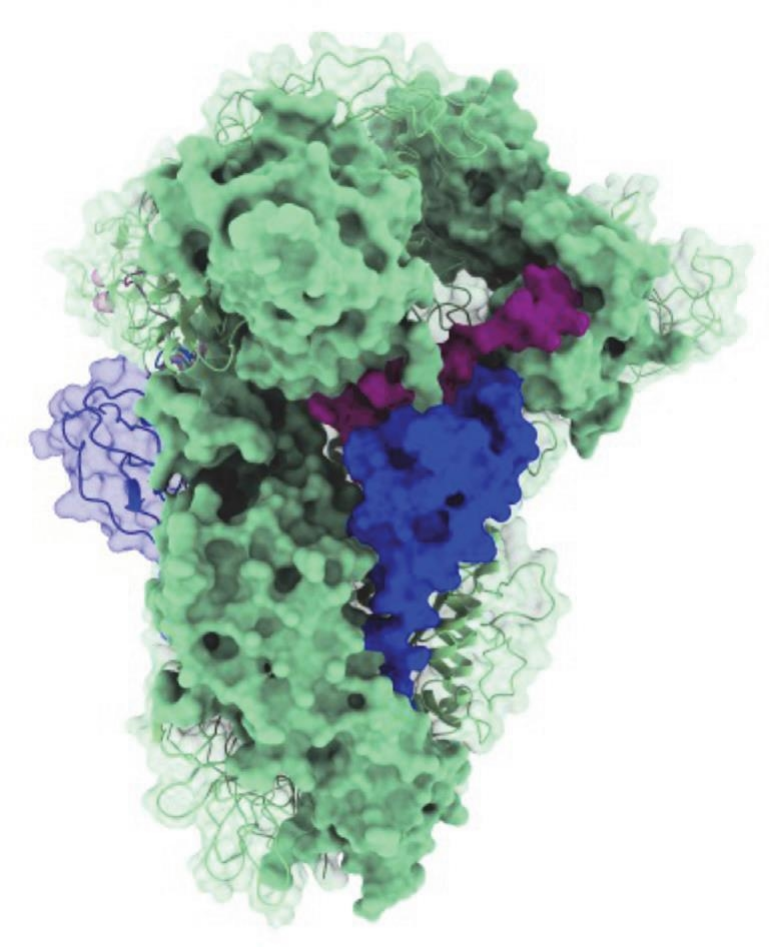
(E)

**Figure:**
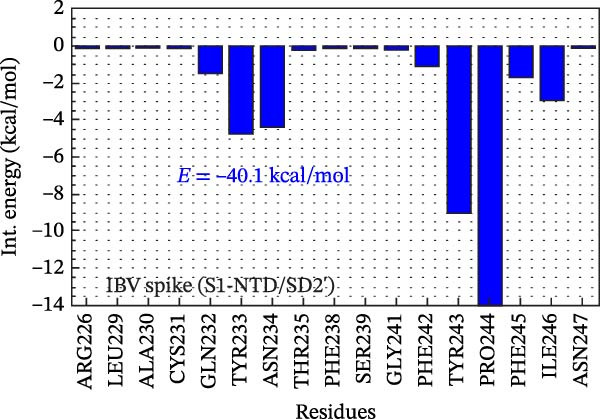
(F)

**Figure:**
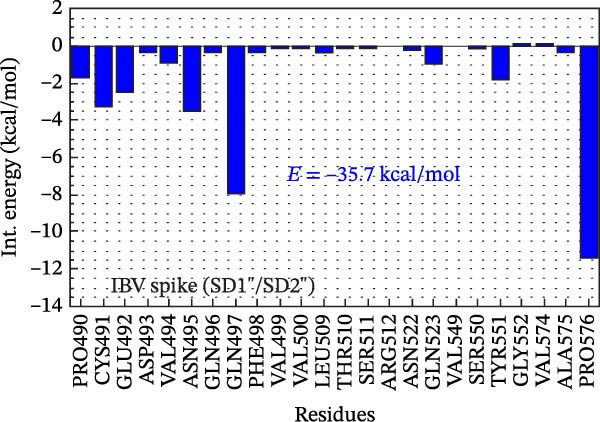
(G)

**Figure:**
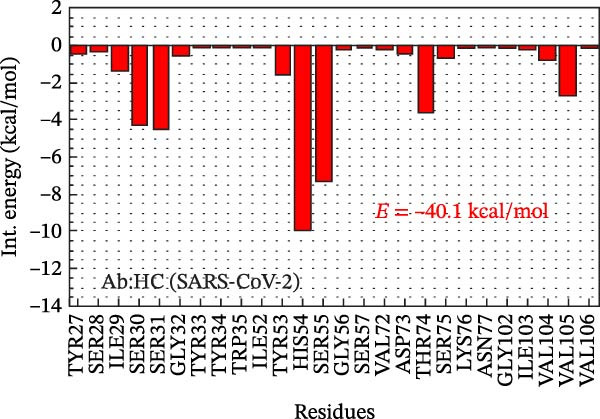
(H)

**Figure:**
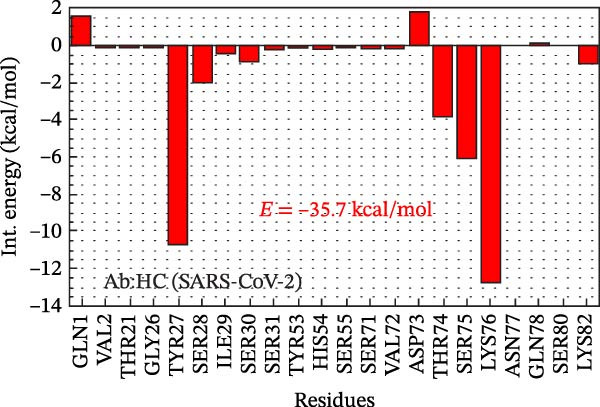
(I)

In Figure 2D, a 2D map of the interaction energies obtained from the molecular fractionation with conjugate cap (MFCC) quantum biochemistry description indicates that only four regions of IBV S and four regions of the antibody P2B‐2F6, designated as hot spots, are responsible for the high‐affinity binding between IBV S and P2B‐2F6. There are only eight interactions among these hot spots, whose energy values are highlighted in Figure 2D, resulting in a cumulative interaction energy of *E*_marked_ = −72.8 kcal/mol. This value represents nearly the entire total binding interaction energy, *E*_TOT_ = −75.8 kcal/mol, between IBV S and P2B‐2F6.

The IBV S hot spot 238–248 shows the strongest interaction energies with the antibody P2B‐2F6, interacting with regions 26–35 (−11.4 kcal/mol), 52–57 (−12.5 kcal/mol), and 72–82 (−5.0 kcal/mol), for a total interaction energy of −28.9 kcal/mol. The second most important IBV S hot spot is the region 490–500, whose interaction energies (*E*_spot’s_) with the regions 26–35 and 72–82 of P2B‐2F6 are −14.4 and −7.4 kcal/mol, respectively. In order of importance, the regions 574–578 (*E*_spot_ = −11.6 kcal/mol) and 226–235 (*E*_spot_ = −10.5 kcal/mol) are the other two hot spots of IBV S. Thus, excepting the residues 490–492 of the SD1″ part of the SD1 subdomain, all the IBV S residues that contribute significantly to the binding to P2B‐2F6 belong to the S1‐NTD domain or the SD2 subdomain (SD2″ and SD2′ parts). In descending order of importance, the hot spots of the antibody P2B‐2F6 are the regions 26–35, 72−82, and 52–57, contributing to the total binding energy between IBV S and P2B‐2F6 with *E*_spot’s_ values of −25.8, −24.0, and −19.2 kcal/mol, respectively.

Figure 2E shows the 3D structure of the IBV spike protein in its closed conformation [14]. The blue‐highlighted region (residues 226–256) includes the S1‐NTD domain (226–237), SD2′ (238–248), and SD1′ (249–256). This area contains the top‐ranking antibody‐binding hot spots 238–248 (SD2′/SD1′) and 226–235 (S1‐NTD). The purple‐highlighted region (490–576, SD1″/SD2″) covers the second and third most important antibody‐binding hot spots, 490–500 and 574–578.

Figure 2F shows the BIRD (binding site, interaction energy, and residue domain) panel, illustrating the binding energy of IBV S residues to the P2B‐2F6 antibody. The binding energy of each IBV S residue is calculated by summing interaction energies within an 8 Å distance from P2B‐2F6 residues. For example, PRO244 has a binding energy of −14 kcal/mol. The 2D map shows each interaction energy (*E*_*ij*_) between an IBV S residue and a P2B‐2F6 residue. The residues 226–256 of IBV S contribute −40.1 kcal/mol to the total binding energy *E*_TOT_ = −75.8 kcal/mol of the complex IBV S/P2F‐2F6. The main contributions to this energy are given by the residues PRO244 and TYR243 of the SD2′ part, whose binding energies to PDF‐2F6 are −14 and −9 kcal/mol, respectively. In the S1‐NTD domain, the residues TYR233 and ASN234 are also relevant to the stabilization of the IBV S/P2B‐2F6 complex, exhibiting binding energies of approximately −4.5 kcal/mol. Furthermore, as shown in Figure 2G, the region 490–576 also plays a crucial role in IBV S/PDB‐2F6 binding, contributing to the total binding energy with the remaining −35.7 kcal/mol. The major contributors to this energy are the residues PRO576 (−11.5 kcal/mol) and GLN497 (−8.0 kcal/mol), located in the SD2″ part. Other relevant residues are ASN465, CYS491, GLU492, and TYR551.

Figure 2H presents the BIRD panel from the antibody’s perspective, highlighting P2B‐2F6 residues with the highest binding energy to IBV S (226‐256 region, 8 Å cutoff). The key residues—HIS54, SER55, SER31, SER30, THR74, and VAL105—show binding energies between −10 and −3 kcal/mol. Figure 2I depicts the binding energies for the P2B‐2F6 residues interacting with the IBV S region 490–576, with LYS76, TYR27, SER75, and THR74 being the most significant, with energies from −13 to −4 kcal/mol. The THR74–SER75–LYS76 triad contributes about −23 kcal/mol to the total binding energy.

### 2.5. Immune Response of Mice Vaccinated With IBV‐H120

Mice that had been immunized with IBV‐H120 developed a humoral and cellular immune response, with the production of polyclonal antibodies (IgG, IgG1, and IgG2a) that were capable of specifically recognizing IBV‐120 through different immunization routes. However, the antibody titers induced by subcutaneous administration with the use of an adjuvant at 28 days postimmunization were observed to be significantly higher than the titers observed in the other routes without adjuvant (Figure 3A).

Figure 3Enzyme‐linked immunosorbent assay (ELISA) with polyclonal antibodies from mice immunized with IBV‐H120 oral and intranasal routes without adjuvant and IBV‐H120 or SARS‐CoV‐2 by the subcutaneous + Al(OH)_3_ (A–D). All serum were used at 1:100 dilution: (A) Detection of serum IgG antibodies 28 days after immunization. (B) Comparison of IgG1 levels at 28 and 45 days after immunization. (C) Comparison of IgG2a levels at 28 and 45 days postimmunization. (D) Evaluation of cross‐reactivity between IBV‐H120 and SARS‐CoV‐2. Negative control group: Serum was collected from the mice before immunization (preimmune). The plates were sensitized with 1 µg of IBV‐H120 vaccine (A–C). The final plate was sensitized with 1 µg of IBV‐H120 vaccine and 1 µg of purified SARS‐CoV‐2 virus (D). Tukey test was used for all experiments. The student’s *t*‐test was also used in conjunction with the Tukey test (B–D). Student’s *t*‐test was used to compare the plates immunized with IBV‐H120 or SARS‐CoV‐2. Tukey’s test was used to evaluate the difference between the different immunization routes. Different letters indicate a statistical difference between the means. Same letters indicate no statistical difference between the means.  ^∗^ *p* < 0.05;  ^∗∗^ *p* < 0.01;  ^∗∗∗^ *p* < 0.001;  ^∗∗∗∗^ *p* < 0.0001. NS, not significant.

**Figure:**
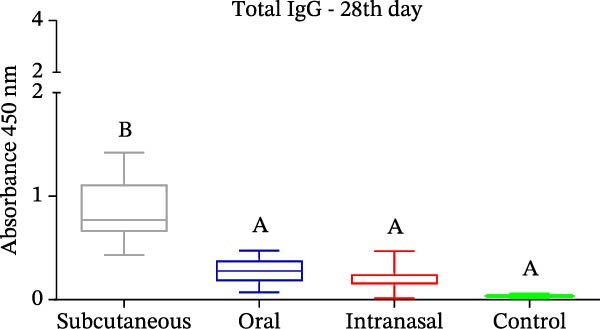
(A)

**Figure:**
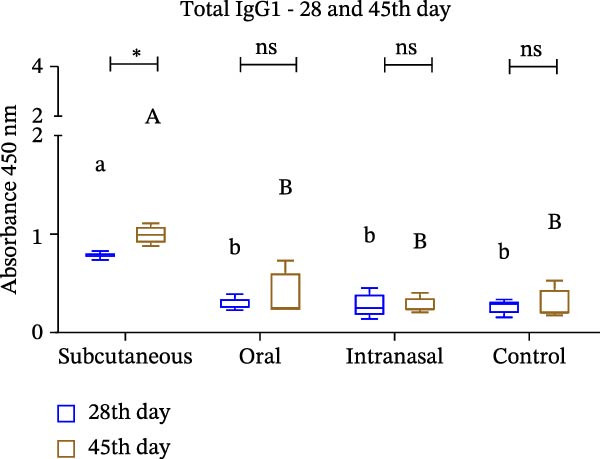
(B)

**Figure:**
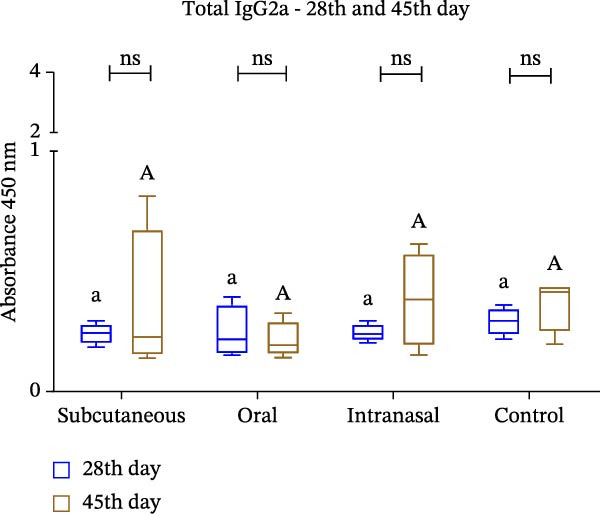
(C)

**Figure:**
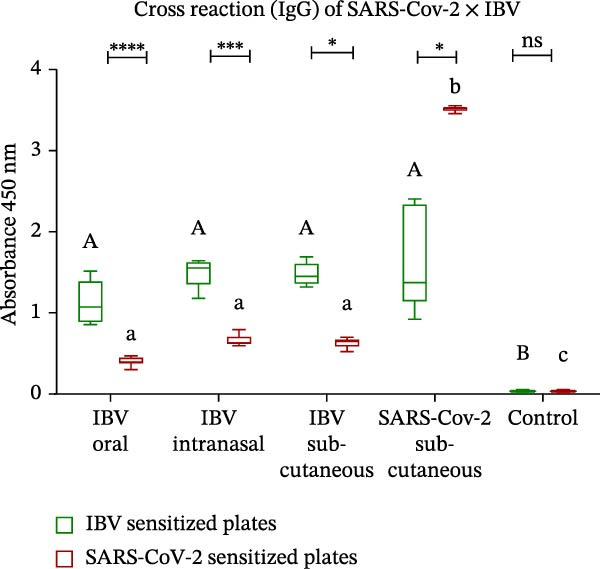
(D)

The same trend was observed for the IgG1 isotype at 28 and 45 days following immunization, as showed in Figure 3B. When we evaluate the presence of IgG2a, we observe no difference between Days 28 and 45, nor between the immunization routes (Figure 3C). The results of the present investigation demonstrate the existence of cross‐reactivity between the IgG‐anti‐IBV antibodies and SARS‐CoV‐2 (Figure 3D).

The PRNT results showed that antibodies neutralized around 94% and 95% of SARS‐CoV‐2 in Swiss mice immunized orally and intranasally, respectively, 28 days postimmunization. It was somewhat unexpected that the antibodies induced by the intranasal route demonstrated the most effective neutralizing capacity against SARS‐CoV‐2 when evaluated (Figure 4A,B), which was statistically significant. The T cell polarization was evaluated using flow cytometry. These analyses revealed an elevation in the proportion of CD119‐positive lymphocytes (Figure 5).

Figure 4(A) Viral neutralization test (PRNT) analysis of SARS‐CoV‐2 neutralization with polyclonal antibodies from mice immunized with 400 viral particles IBV‐H120 vaccinal by the subcutaneous + Al(OH)_3_, oral and intranasal routes (in the absence of adjuvant). Negative control group: was used preimmune serum not reactive for SARS‐CoV‐2. Tukey’s test was used to evaluate the difference between the different immunization routes and negative control. Different letters indicate a statistical difference between the means. (B) ANOVA revealed significant differences (*p* < 0.05) in plaque formation percentages among immunization routes at higher serum dilutions (1/16–1/1024), compared with the negative control. Effect size analyses (*η* ^2^ and Cohen’s *d*) indicated that, although ANOVA and Tukey’s post hoc tests did not confirm statistically significant differences, notable trends were observed: effect sizes were small at lower dilutions, but increased to moderate and large magnitudes at higher dilutions. Importantly, the nasal route consistently demonstrated greater robustness, suggesting enhanced durability of the immune response under extreme conditions. These findings highlight that, even in the absence of formal statistical significance, the nasal route may confer superior immunological resilience. Same letters indicate no statistical difference between the means.

**Figure:**
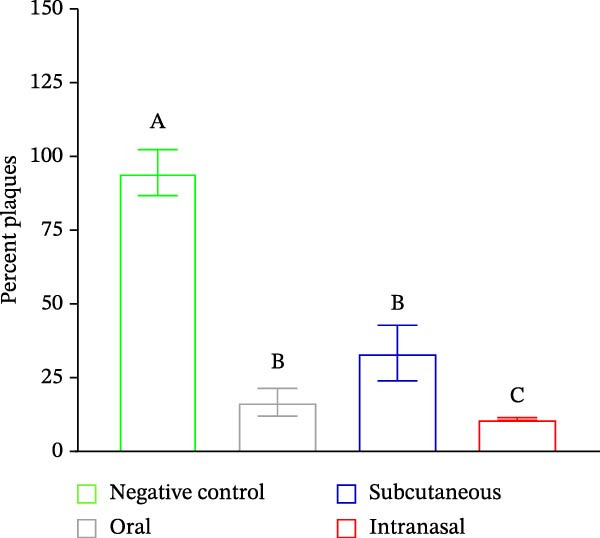
(A)

**Figure:**
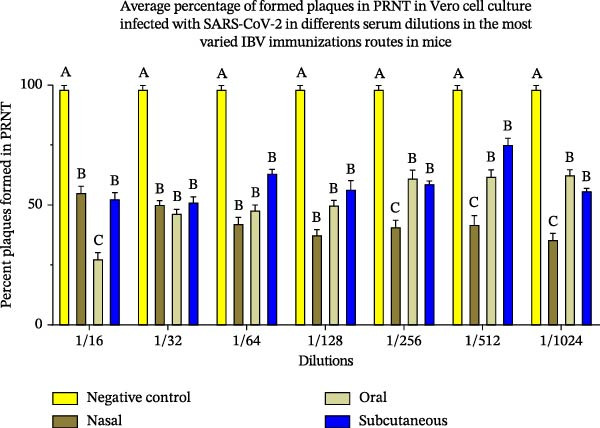
(B)

Figure 5Flow cytometry analysis of T cell activation in mice. (A) Median fluorescence intensity (MFI) of IFN‐γ^+^ lymphocytes (CD3^+^CD8^+^ CD19^−^) in treated and control groups. (B) Frequency (%) of IFN‐γ^+^ lymphocytes (CD3^+^CD8^+^CD19^−^) relative to the parent population. (C) Analysis of CD119^+^ lymphocyte subpopulation levels in mice immunized intranasally with the IBV‐H120 vaccine compared with saline‐immunized control mice. The flow cytometry gating strategy used in this analysis is shown in Figure 5. Data are presented as mean ± standard deviation (SD). Each experimental group consisted of seven independent biological samples (*N* = 7). The results are representative of two independent experiments performed under identical conditions. A negative control consisted of cell cultures with virus added. Statistical analysis was performed using the Mann–Whitney test. Different uppercase or lowercase letters indicate statistically significant differences within the same group. Statistical significance was indicated as follows: *p* < 0.0001.

**Figure:**
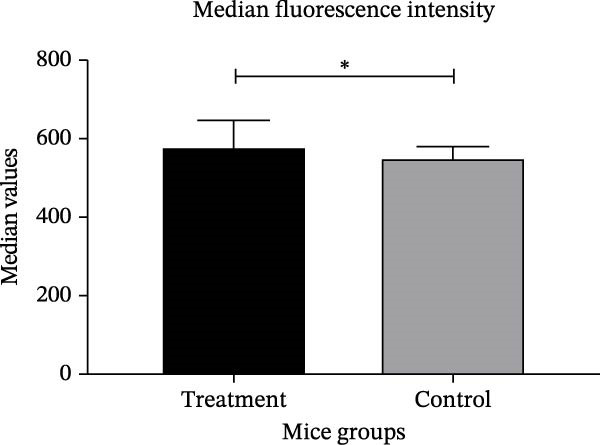
(A)

**Figure:**
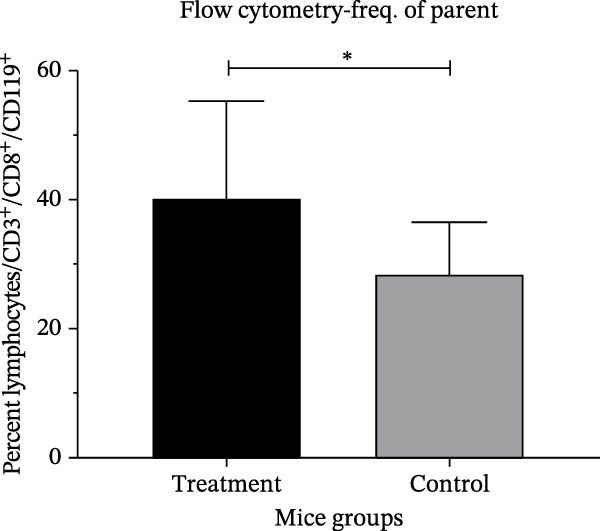
(B)

**Figure:**
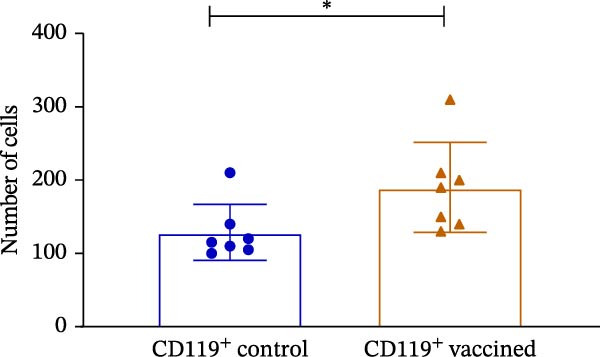
(C)

### 2.6. Histopathological Analysis

**Figure 7:**
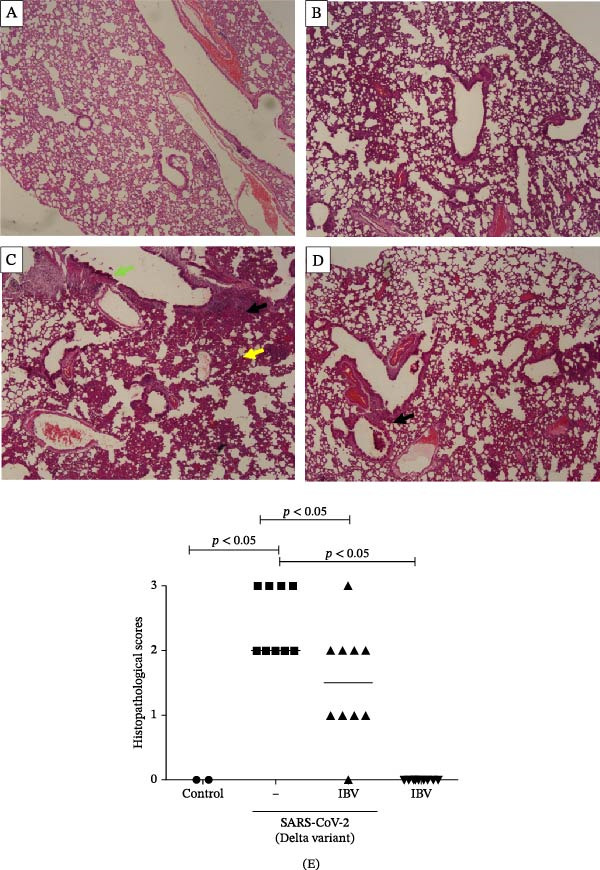
Histopathologic analysis of the lungs of Syran hamsters vaccinated intranasally with IBV‐H120 and challenged with SARS‐CoV‐2 (2 × 10^3^, viral particles, Delta). (A) Healthy; (B) intranasal IBV‐H120; (C) placebo; (D) intranasal IBV‐H120. The groups showed in Subfigures (C, D) were challenged by intranasal administration of 2 × 10^3^ SARS‐CoV‐2 (Delta strain) viral particles. Lung sections (5 μm) were obtained for hematoxylin–eosin staining (H&E) and analysis under light microscopy (magnification 200x). Subfigure (E) depicts the score values for tissue injury are expressed as the median and range. The black arrow indicates a peribronchial inflammatory infiltration; the yellow arrow denotes the consolidation of the alveolar airspaces; the green arrow points out a reactive epithelial degeneration. Scale bar = 100 μm. The score values for tissue injury are expressed as the median and range. Data were analyzed by the Kruskal–Wallis, followed by Dunn’s test.

Histopathological analysis of the lungs is presented in Figures 6 and 7 in high magnification (200x). As shown in Figures 6C and 7C, SARS‐CoV‐2 infection induced significant lung injury. It was characterized by peribronchial inflammatory infiltration, consolidation of the alveolar airspaces, and reactive epithelial degeneration, differing from normal lungs of noninfected hamsters (Figures 6A and 7A). Additionally, intranasal IBV‐H120 treatment (Figures 6D and 7D) attenuated the tissue damage mainly characterized by mild peribronchial inflammatory infiltration. Conversely, subcutaneous IBV‐H120 administration did not protect from SARS‐CoV‐2 infection (Figures 6B and 7B). The analysis of the lungs (Figures 6E and 7E) by scores indicated moderate to severe damage in the SARS‐CoV‐2 infected hamsters (2.5), contrasting from normal tissue of noninfected animals (0 [0–0]; *p*  < 0.05). The score analysis of lungs obtained from hamsters treated with intranasal IBV‐H120 showed mild to moderate alterations (1 [1,2], *p* = 0.05 vs. the SARS‐CoV‐2 group). Figures S4 and S5 display identical photomicrographs at a low magnification (40x).

Figure 6Histopathologic analysis of the lungs of Syran hamsters vaccinated intranasally with IBV‐H120 and challenged with SARS‐CoV‐2 (2 × 10^3^, viral particles, Wuhan). (A) Healthy; (B) subcutaneous IBV‐H120; (C) placebo; (D) intranasal IBV‐H120. The groups showed in Subfigures (B–D) were challenged by intranasal administration of 2 × 10^3^ SARS‐CoV‐2 (Wuhan strain) viral particles. Lung sections (5 μm) were obtained for hematoxylin–eosin staining (H&E) and analysis under light microscopy (magnification 200x). Subfigure (E) depicts the score values for tissue injury are expressed as the median and range. The black arrow indicates a peribronchial inflammatory infiltration; the yellow arrow denotes the consolidation of the alveolar airspaces; the green arrow points out a reactive epithelial degeneration. Scale bar = 100 μm. The score values for tissue injury are expressed as the median and range. Data were analyzed by the Kruskal–Wallis, followed by Dunn’s test.

**Figure:**
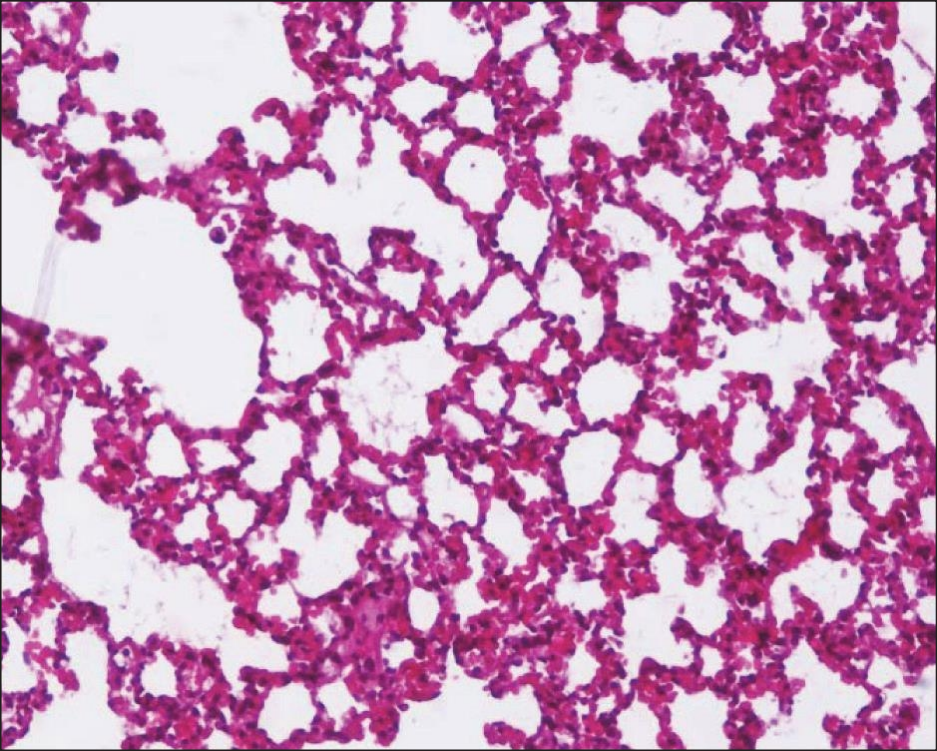
(A)

**Figure:**
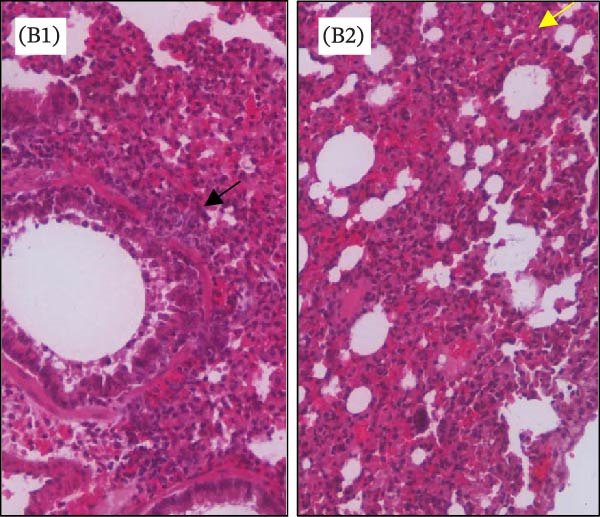
(B)

**Figure:**
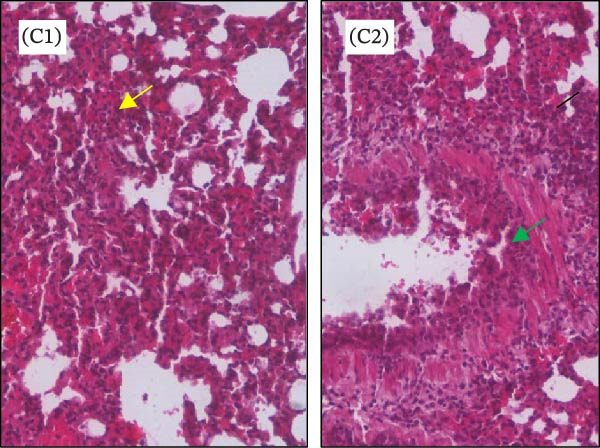
(C)

**Figure:**
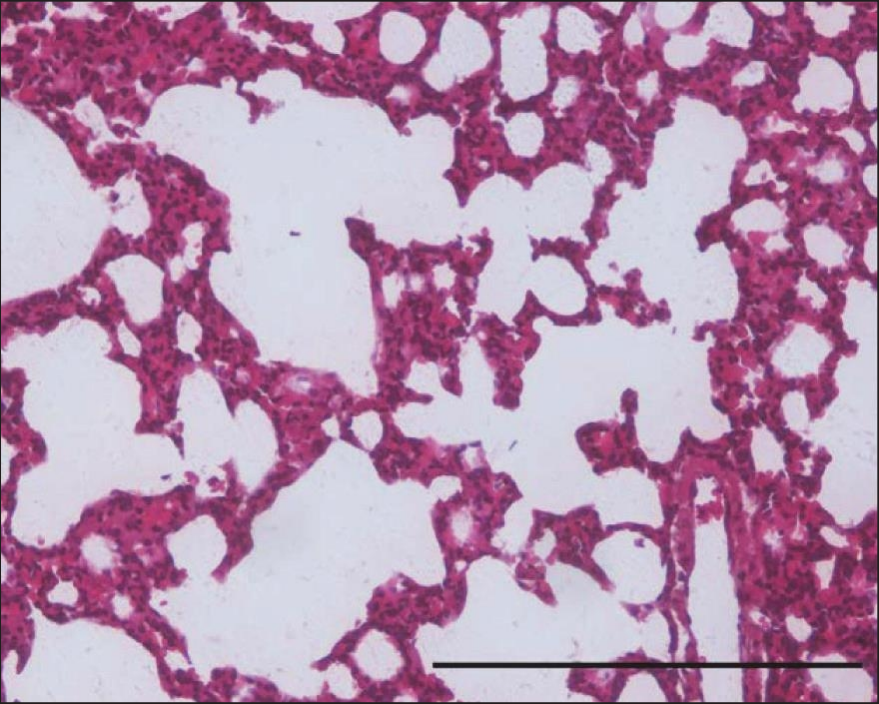
(D)

**Figure:**
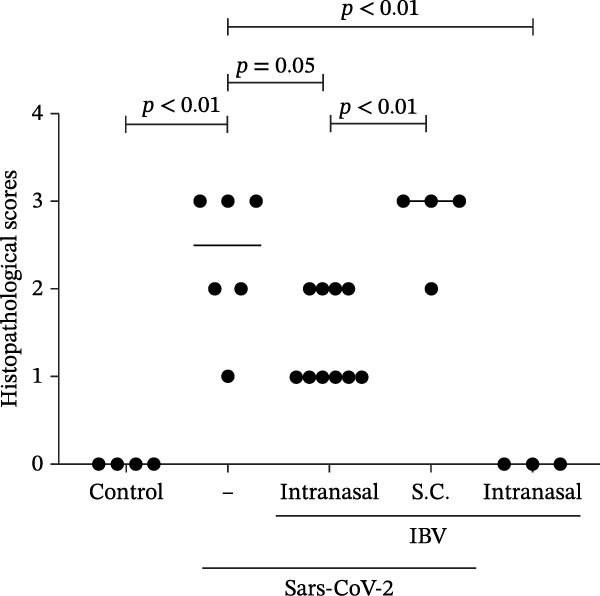
(E)

## 3. Discussion

Our research evaluated the use of the avian IBV, H120 variant, as a potential candidate for the development of a vaccine against SARS‐CoV‐2. During the pandemic, it was observed that workers administering the IBV‐H120 vaccine to birds on a farm near Fortaleza were not infected with the COVID‐19 virus. They continued working normally despite having contact with family members and other workers from the same farm who had contracted the disease. Twelve oropharyngeal and serum samples were collected: two from the administration department and 10 from the vaccinators, along with 10 prepandemic control serum samples. All vaccine handlers tested negative for PCR. On the other hand, two administration workers tested positive for COVID‐19 and were excluded from the study. In chemiluminescence assays, we observed that only 50% of poultry farm workers had serum IgG antibodies that specifically reacted with the S1 protein of SARS‐CoV‐2. This result is consistent with Maciola et al. [15], who reported a modest proportion of IgG specific to the Spike protein.

The same result was observed in indirect ELISA, which showed a 50% (5/10) detection of IgG that specifically recognized purified SARS‐CoV‐2 from cell culture. However, the detection rate of serum IgG reactive with the IBV‐H120 vaccine was only 20% (2/10). On the other hand, when evaluating immunoglobulin isotypes (IgA and IgM), we found the presence of specific serum IgA for both viruses (IBV and SARS‐CoV‐2) in 90% (9/10) of the workers and specific IgM in 70% (7/10) for SARS‐CoV‐2 and 60% (6/10) for IBV‐H120 (Figure S2A, B).

The higher percentage of serum IgA is consistent with Padoan et al. [16], who found elevated levels of IgA in patients with COVID‐19 compared to IgM levels. At the same time, specific reactions to either virus were observed in the control group. These results suggest a cross‐reactivity between IBV‐H120 and SARS‐CoV‐2. Similar findings were reported by Ardicli et al. [11] with variants of IBV other than the H120 strain, who described the presence of anti‐IBV IgG antibodies in the serum of poultry farm workers that specifically reacted with S1‐SARS‐CoV‐2. Furthermore, Ardicli et al. [11], who highlighted a positive correlation between specific IgG for IBV and specific IgG for SARS‐CoV‐2, which may indicate the presence of cross‐reactive antibodies.

The antibodies of poultry farm workers neutralized around 53% and 94% of SARS‐CoV‐2, while the prepandemic control samples did not show any neutralization effect (Figure S2C). Viral neutralization levels showed independence of antibody titers (IgM, IgA, and IgG; Figure S1B).

Although the sample size is small, these results suggest that serum IgA may play a role in controlling SARS‐CoV‐2. While serum IgA is the second most abundant antibody after IgG, there is limited evidence on its neutralizing function against SARS‐CoV‐2, as most studies focus on mucosal IgA. Few studies, such as those by Guo et al. [17] and Padoan et al. [16], have detected specific serum IgA for SARS‐CoV‐2. Leong and Ding [18] also highlight the importance of serum IgA in controlling COVID‐19, but further studies with larger cohorts are needed [19].

A bioinformatics analysis was conducted to gain a more comprehensive understanding of the phenomenon of cross‐reactivity between SARS‐CoV‐2 and IBV. The in silico analysis revealed the relevant interactions of the IBV S/P2B‐2F6 complex, allowing us to identify the key residues for the binding of IBV S to the antibody P2B‐2F6. For example, we observed the favorable contribution of the residue SER30 (antibody) to the high interaction between IBV S and the antibody P2B‐2F6. As shown in Figure 2F,G, the binding energies of SER30 to the IBV S regions 226–256 (S1‐NTD/SD2′) and 409–576 (SD1″/SD2″) are −4.5 and −1.0 kcal/mol, respectively, resulting in a total binding energy to IBV S of −5.5 kcal/mol. This result is consistent with the findings of Liu et al. [20], which also evaluated the molecular interaction between the key residue N501 in the SARS‐CoV‐2‐RBD epitope and the screening antibody B38, revealing a strong attractive energy of −3.32 kcal/mol for the SER30 residue of B38.

Additionally, the X‐ray resolved structure of the SARS‐CoV‐2 induced human neutralizing antibody (P2B‐2F6) complexed with SARS‐CoV‐2‐RBD, as described by Ju et al. [21], revealed the paratope consisting of 11 heavy chain residues (TYR27, SER28, SER30, SER31, TYR33, HIS54, GLY102, ILE103, VAL105, VAL106, and PRO107) and three light chain residues (GLY31, TYR32, and ASN33), acting as the structural basis for RBD interactions.

Remarkably, in our quantum biochemistry analysis of the P2B‐2F6 antibody complexed with IBV S, the heavy chain paratope residues, except for SER31 and PRO107, align with those identified by Ju et al. [21]. The highest affinity interactions with IBV S were found in TYR27, SER28, SER30, HIS54, and VAL105. Additionally, despite low binding energies, TYR33, GLY102, ILE103, and VAL106 were included in the total binding energy calculation for IBV S to P2B‐2F6 (Figure 2H,I). These findings indicate that IBV may stimulate the production of neutralizing antibodies against SARS‐CoV‐2, akin to the P2B‐2F6 antibody, supporting previous reports and correlating with subsequent animal experiment results.

After analyzing serum samples from poultry vaccinators and other groups, we assessed the immune response in mice. Using indirect ELISA and flow cytometry, we found that IBV‐H120 vaccination induced a humoral immune response with IgG, IgG1, and IgG2a production via different immunization routes. This is significant as it represents the first demonstration of an experimentally induced immune response by attenuated IBV‐H120 in mammals.

The subcutaneous administration of the vaccine with adjuvant elicited a stronger immune response in mice, evidenced by higher titers of IgG, IgG1, and IgG2a antibodies (Figure 4). These differences were significant compared to groups immunized via oral and intranasal routes without adjuvant. Importantly, all antibodies specifically recognized both IBV‐H120 and SARS‐CoV‐2, demonstrating cross‐reactivity between these viruses. Additionally, the observed IgG isotype switching suggests effective activation of CD4^+^ T cells, which play a central role in the adaptive immune response.

Upon activation of the T cell receptor and co‐stimulation by antigen‐presenting cells, naïve CD4^+^ T cells differentiate into one of several lineages of helper T cell subtypes [22]. Thus, B‐cell responses are largely dependent on extracellular cytokines, which have a strong impact on subsequent B‐cell responses [23]. Cytokines produced by helper T cells are essential for the body’s defense against viruses [24]. IFN‐γ and IL‐4 are characteristically secreted during Th1 and Th2 responses [25, 26].

In contrast, serum from subcutaneously immunized animals showed lower neutralization. The PRNT results showed that antibodies neutralized around 94% and 95% of SARS‐CoV‐2 in Swiss mice immunized orally and intranasally, respectively, 28 days postimmunization. These findings suggest that not all antibodies induced by the subcutaneous route neutralize, highlighting the importance of immunization routes in vaccine development. While the subcutaneous route is commonly used to induce circulating antibodies, it often requires adjuvants for an effective immune response, which poses challenges due to the limited number of adjuvants suitable for human use [27].

Given the immune response induced in mice immunized via oral and intranasal routes without adjuvant, which produced neutralizing antibodies against SARS‐CoV‐2, the intranasal route was chosen for further study. This route offers several advantages: it mirrors the pathogen’s entry, is noninvasive, natural, and does not require adjuvants [28]. This would facilitate potential mass vaccination efforts and increase the public’s acceptance of the vaccine. According to Chavda et al. [29], among the numerous mucosal immunization routes, the intranasal route is one of the most studied alternatives due to its established commercial configuration.

In conclusion, the intranasal route represents a promising approach for the development of vaccines against SARS‐CoV‐2, given that the nasal mucosa is often the primary site of infection [29]. Intramuscular administration induces strong production of serum IgG, which is thought to be protective of the lower respiratory tract but does not induce the epithelial cell IgA responses necessary for protection of the upper respiratory tract [27].

To better understand the cellular immune response induced by IBV‐H120, spleen cells from vaccinated mice were analyzed via flow cytometry for T lymphocyte markers. Compared to controls, an increase in CD8^+^, T‐bet, and interferon‐γ receptor expression was observed, with only interferon‐γ showing significant differences. This suggests a tendency for antiviral response activation.

Previous research by Ariaans et al. [30] showed in vitro that stimulation with IBV resulted in the production of IFN‐γ by splenocytes cells in birds. However, the same authors were unable to stimulate IFN‐γ in mammalian cells using cells. Our results are the first report of induction of interferon‐γ production by IBV‐H120 in mammals in vivo. Aleebrahim‐Dehkordi et al. [24] report that TNF‐α and IFN‐γ synergistically activate macrophages and induce antiviral responses via lung epithelial receptors. The T cell activation and interferon production observed in our study with IBV‐H120 is significant, as T cell activity correlates with less severe SARS‐CoV‐2 infection, highlighting its key role in controlling and treating the disease. The early, potent IFN response is crucial for fighting viral infections [31].

Besides inducing an antiviral state in cells, IFN‐γ plays a key role in modulating host defense mechanisms, including activating macrophages, natural killer cells, and inducing MHC Class I and II molecules [32, 33]. The alpha chain of the IFN‐γ receptor (IFN‐γR1/CD119) was the first receptor component identified. IFNs exhibit various antiviral effects, such as activating cytotoxic T cells, inhibiting viral mRNA translation, degrading viral RNA, editing RNA, and modulating nitric oxide synthesis [32].

To evaluate whether the immune response induced by IBV‐H120 confers protection against SARS‐CoV‐2, vaccination trials were conducted in hamsters *Mesocricetus auratus*, followed by a challenge with a high dose of live virus. Intranasal vaccination with IBV‐H120 reduced lung damage in against a high viral load (2 × 10^3^ TCID50) of the Wuhan SARS‐CoV‐2 strain and also infection with the Delta variant. Lung histopathology in the intranasal group was comparable to that of healthy controls. These findings are consistent with Langel et al. [34], who observed similar protection in hamsters vaccinated with an Adenovirus Type 5 vaccine expressing the SARS‐CoV‐2 spike protein.

This experimental model is widely used in studies involving SARS‐CoV‐2, as it expresses an ACE2 receptor highly similar to humans. This similarity makes these animals naturally susceptible to SARS‐CoV‐2 infection. Following infection, they exhibit clinical, virological, and pathological features closely resembling the more severe forms of COVID‐19 observed in humans [35, 36]. Due to these characteristics, we used the Syrian hamster (*Mesocricetus auratus*) model to evaluate whether the immune response induced by IBV could trigger cross‐reactivity and provide protection against SARS‐CoV‐2.

On the other hand, the animals that were vaccinated by subcutaneous injection + aluminum hydroxide adjuvant were not protected. Subcutaneously vaccinated hamsters infected with the virus developed severe pneumonia with peri bronchial infiltrate and consolidation, a process associated with alveolar closure. This impairs alveolar gas exchange, leading to shortness of breath and epithelial damage, airway compromise, septal thickening, and inflammatory infiltrates. These symptoms resemble those seen in humans with SARS‐CoV‐2 infection [37, 38].

Despite the promising results, this study has some limitations that should be acknowledged. Future investigations using additional immunological and molecular markers specific to hamsters are needed to more precisely elucidate the mechanisms underlying the cross‐reactivity and protection observed between IBV‐H120 and SARS‐CoV‐2.

## 4. Conclusion

Vaccination has been widely recognized as the most effective method to prevent, control, and reduce morbidity and mortality caused by the novel coronavirus (COVID‐19). Several vaccines have been rapidly developed and licensed for this purpose. Nevertheless, the search for safer, low‐cost, and especially easy‐to‐store vaccines continues. The findings of this study indicate that the IBV‐H120 induces cross‐reactivity with SARS‐CoV‐2 via activation of innate immune and adaptive immune mechanisms, leading to production of neutralizing antibodies against the virus. Moreover, the study demonstrates that IBV‐H120 reduceded lung damage in hamsters challenged with the Wuhan and Delta variants. However, more studies need to be conducted to measure the protective immune response induced by IBV‐H120 against SARS‐CoV‐2.

## 5. Methods

### 5.1. Ethics Approval and Consent to Participate

The participants who worked on the poultry farm signed an informed consent form that was approved by the Ethics and Research Committee of the Federal University of Ceará/Walter Cantídio Hospital (UFC/HUWC Number 5.177.381 and CAAE Number 53156521.2.0000.5045). Animal experiments were conducted in accordance with protocols reviewed and approved by the Animal Research Ethics Committee of UECE, Brazil, with protocol number 20620/2020.

### 5.2. Isolation and Identification of the SARS‐CoV‐2

The SARS‐CoV‐2 was isolated from clinical samples obtained from the Hematology and Hemotherapy Center of Ceará (HEMOCE). Viral isolation was performed following the method described by Harcourt et al. [39] with modifications. The clinical sample was filter‐sterilized (0.22 µM) and inoculated into a flask containing a monolayer of Vero E6 cells with 90%–100% confluence. The flask was incubated for 1 h at 37°C with gentle agitation. Following incubation, the medium was replaced with L‐15 medium containing 2% FBS and 1% penicillin/streptomycin antibiotics. The cells were then incubated at 37°C for viral propagation and monitored daily for cytopathic effects. Virus titration was performed using the “plaque‐forming units” (PFUs) technique, following the protocol previously described by Mendoza et al. [40]. Viral sequencing was conducted using the CovidSeq kit (Illumina).

The isolated viruses were identified as the variant B.1.212 (Wuhan strain) and A.Y.99.2 (Delta strain) which were circulating in the state of Ceará/Brazil during the collection period [41]. The viral title of the Delta variant was determined to be 7.8 × 10^4^ viruses per milliliter (mL) using the PFU technique. For the Wuhan variant, the titer was found to be 1.1 × 10^5^ PFU/mL. Viral sequencing was conducted using the CovidSeq kit (Illumina) and deposited in GISAID (https://gisaid.org/) with the numbers EPI_ISL_19324956 [42] and EPI_SET_240409kz [43].

### 5.3. Purification of SARS‐CoV‐2 and IBV Strain H120 Used in the Immunoenzymatic Assays

The SARS‐CoV‐2 isolate, obtained as described in the previous section, was purified following the method described by Florindo et al. [44], with minor modifications. The virus was propagated in Vero E6 cells and inactivated in a water bath at 60°C for 10 min. The viral suspension was then diluted in 0.1 M potassium phosphate buffer (pH 7.5) containing 0.5% sodium sulfite as an antioxidant in a 1:2 (*v*/*v*) ratio. This mixture was gently agitated for 2 h at 20°C and centrifuged at 3000 × *g* for 10 min at 4°C. The pellet was discarded, and polyethylene glycol (PEG) 6000 and NaCl were added to the supernatant to final concentrations of 8% and 4%, respectively. The solution was gently mixed on ice for 2 h to ensure homogenization, followed by centrifugation at 10,000 × *g* for 10 min. The supernatant was discarded, and the pellet was resuspended in 0.01 M phosphate buffer (pH 7.5). After gentle agitation for 1 h, the suspension was centrifuged at 12,000 × *g* for 10 min. The pellet was discarded, and the supernatant containing the virus was subjected to three additional cycles of PEG precipitation as previously described. After the final PEG precipitation step, the supernatant was ultracentrifuged at 120,000 × *g* for 2 h. The resulting supernatant was discarded, and the pellet was resuspended in 0.01 M phosphate buffer (pH 7.5) and stored at −20°C for subsequent use in immunoenzymatic assays, following the methodology of Marques et al. [45] and Herazo et al. [46].

#### 5.3.1. IBV Strain H120

Samples of the avian IBV strain H120 were obtained by reconstituting the commercial H120 Mass‐I/Zoetis vaccine in distilled water and immediately using it, following the attenuated virus titer specified by the manufacturer.

#### 5.3.2. In Vitro Infection of Mammalian Cell Lines

The replication capacity of the attenuated IBV‐H120 vaccine strain in mammalian cells was evaluated through in vitro infection assays. The following cell lines were used: RIN‐5F (CRL‐2058), Vero (CCL‐81), HEK‐293 (CRL‐1573), Calu‐3 (HTB‐55), and HD11 (CRL‐1745). Cells were cultured under lineage‐specific conditions until approximately 80% confluence. Cells were infected with the IBV‐H120 strain, and culture supernatants and cell pellets were collected at 24, 48, 72, and 96 h postinfection, followed by daily sampling up to 11 days, except for HEK‐293 cells, which were monitored up to 96 h due to cell death. Viral RNA was detected by RT‐qPCR using RNA extracted from supernatants and cell pellets, followed by cDNA synthesis and amplification according to standardized protocols.

### 5.4. Collection and Analysis of Human Samples

Blood samples and oropharyngeal swabs (*n* = 12) were collected from poultry farm workers in Ceará, Brazil, between May and June 2021. Of these, 10 individuals were directly involved in administering the live attenuated IBV vaccine (H120, Mass‐I/Zoetis) through aerosol spraying in poultry houses, while two workers from the coordination sector, who had no direct contact with poultry or the vaccine, were also included. The latter tested positive by PCR and were excluded from subsequent analyses. None of the employees wore masks during vaccine handling. Thus, the final sample size was *N* = 10.

Blood samples were collected in nonanticoagulant tubes, which were then hermetically sealed and arranged in transport racks. The samples were stored at 4°C. Afterward, the samples were centrifuged at 3000 × *g*, and the serum obtained was transferred to plastic microtubes and frozen at −20°C.

Oropharyngeal samples were collected using swabs and immediately placed in standard commercial tubes. Subsequently, the tubes were hermetically sealed, arranged in transport racks, and stored at 4°C. After collection, the samples were transported to the Laboratory of Biotechnology and Molecular Biology at the State University of Ceará (UECE) for further analysis. The oropharyngeal samples underwent real‐time RT‐qPCR analysis using the Molecular SARS‐CoV‐2 (E/RP) kit (Bio‐Manguinhos/Fiocruz, Brazil).

As negative controls, serum samples obtained from the prepandemic were used (*n* = 10). These samples were obtained from the serum banks at HEMOCE no‐reactive to the S and N proteins using the SARS‐CoV‐2 IgG II Quant kit (Abbott).

#### 5.4.1. ELISA

An indirect ELISA was used to assess poultry farm workers’ serum (*n* = 10) IgG, IgM, and IgA antibodies against IBV and SARS‐CoV‐2. Microwell plates (U96‐POLYSORP‐NUNC) were coated with IBV (H120) or purified SARS‐CoV‐2, diluted in sodium carbonate buffer (50 mM, pH 9.6, 1 µg/well), and incubated at 4°C for 12 h. After washing with phosphate‐buffered saline containing Tween (PBS‐T) and blocking (2 h at 37°C), 100 µL of diluted serum (1:100 in PBS) was applied in duplicate and incubated for 2 h at 37°C. Following another wash, 100 µL of anti‐IgA (INVITROGEN‐055220), anti‐IgG (INVITROGEN‐H10307), or anti‐IgM (SIGMA‐A0420) conjugated with peroxidase (diluted 1:5000) was added and incubated (2 h at 37°C). TMB (3,3′,5,5′ tetramethylbenzidine) solution (100 µL) was added, and plates were incubated for 20 min in the dark. Absorbance at 650 nm was measured, and cut‐off values were determined as the mean absorbance plus two standard deviations of the negative controls.

#### 5.4.2. Chemiluminescence Immunoassay (CLIA)

The detection of IgG antibodies against the RBD of the S1 subunit of the SARS‐CoV‐2 spike protein was performed using the SARS‐CoV‐2 IgG II Quant kit (Abbott), based on a CLIA. Serum samples were incubated with paramagnetic microparticles coated with viral antigen. After washing, an acridine‐labeled anti‐human IgG conjugate was added. The chemiluminescent signal, generated by successive reactions with pretrigger and trigger solutions, was measured using the ARCHITECT i2000SR analyzer (Abbott).

### 5.5. Plaque Reduction Neutralization Test (PRNT)

To assess the neutralizing potential of antibodies in serum samples of poultry farm workers, each sample was heated at 56°C for 30 min to inactivate the complement system. Then, 60 PFU of SARS‐CoV‐2 were incubated with diluted serum (1:100) for 1 h at 37°C. The virus–serum mixture was added to Vero E6 cells, incubated for 2 h, and then overlaid with 1.5% carboxymethylcellulose. After 3 days at 37°C, formaldehyde was added, followed by staining with crystal violet. Plaques were counted using ImageJ, and neutralization was calculated based on the formula [47]

Plaque reduction neutralization test formula:Sample×100/positive control−100.

### 5.6. In Silico Methods: Target Editing, Docking, Molecular Dynamics, MFCC, and Quantum Biochemistry Calculations

The protein targets were obtained from the Protein Data Bank (PDB), with structure 6CV0 for the IBV spike protein [14] and structure 7BWJ for the antibody P2B‐2F6, produced against the RBD of SARS‐CoV‐2 [21]. P2B‐2F6 is a neutralizing monoclonal antibody against SARS‐CoV‐2 that specifically targets the RBD, blocking viral entry into host cells by interacting with both the up and down conformations of this domain. In addition, P2B‐2F6 exhibits cross‐reactivity with the spike proteins of SARS‐CoV and MERS‐CoV, although it lacks neutralizing activity against these viruses. Nevertheless, given the absence of human antibodies directed against IBV, P2B‐2F6 was considered a potential candidate for cross‐reactivity analysis with this virus.

In selecting this antibody for computational modeling, the first important criterion considered was its lack of neutralizing activity against the SARS‐CoV and MERS‐CoV viruses [21]. It was also taken into account that this antibody belongs to a class that interacts predominantly with a region distinct from the interface between the RBD of the SARS‐CoV‐2 spike protein and the human ACE2 receptor, even though some residues within the RBD participate in this interaction [48]. It is worth noting that the immune evasion of SARS‐CoV‐2 is strongly influenced by mutations in the RBD [49], therefore, antibodies recognizing epitopes located in alternative domains are particularly desirable. In this context, the computational analysis focused on a direct comparison between the IBV and SARS‐CoV‐2 viruses.

The systems were prepared by the missing residues correction [50], protonation adjustment [51], and selecting the region of interest of the IBV S protein [52]. In addition, refinement by the Patchdock server [53], docking on the FireDock server [54], and protein‐antibody binding affinity was evaluated by the PRODIGY server (*ΔG*) [55]. From the pose obtained in the docking, molecular dynamics was carried out using the GROMACS v2021 software [56]. The quantum biochemistry calculations for the IBV S/P2B‐2F6 interactions were evaluated using an adaptation of the MFCCs [57] within the density functional theory (DFT) calculations that were performed using DMol [3] code [58]. The detailed parameters for these procedures are available in the Supporting Information.

### 5.7. Serological Analysis and Plaque Reduction Neutralization Test in Swiss Mice

Male Swiss mice (7–8 weeks old, 20–30 g) were obtained from the UECE Central Animal Facility. The animals were acclimatized in polypropylene cages with water and feed provided ad libitum at 25 ± 2°C under a 12‐h light–dark cycle. They were randomly divided into groups (*n* = 10) and immunized following the methods described by Sánchez‐Felipe et al. [59], as per Table 1. Blood samples were collected on Days 0 (preimmune), 28, and 45, centrifuged, and serum was stored at –20°C for analysis. Serological assays were performed as described in Section 5.4.1, but using secondary anti‐mouse antibodies conjugated to peroxidase for the detection of IgG (A9917, Sigma–Aldrich), IgG1 (A10551, Life Technologies) and IgG2a (A10685, Sigma–Aldrich). PNRTs were conducted as described in Section 5.5.

**Table 1:** Mouse experimental groups and immunization protocol.

Immunization route/group	Intranasal IBV (*n* = 10 animals)	Oral IBV (*n* = 10 animals)	Subcutaneous IBV (*n* = 10 animals)	Subcutaneous SARS‐CoV‐2 (*n* = 10 animals)	Control (*n* = 10 animals)
Immunogen/volume	IBV‐H120 strain (400 viral particles)/25 µL	IBV‐H120 strain (400 viral particles)/25 µL	IBV‐H120 strain (400 viral particles)/100 µL	Inactivated SARS‐CoV‐2 (400 viral particles)/100 µL	PBS
Adjuvant	None	None	Aluminum hydroxide (0.05 M)	Aluminum hydroxide (0.05 M)	None
Immunization timing	First immunization (Day 0) and booster dose (Day 15^th^) and serum collection 28 and 45 days after the start of immunization				

### 5.8. Flow Cytometry

#### 5.8.1. Immunization of Mice With IBV‐H120

Fourteen 8‐week‐old male Swiss mice were divided into two groups (*n* = 7) to assess the cellular response to the attenuated vaccine virus of the commercial IBV‐H120 vaccine. The treatment group was immunized intranasally with the IBV‐H120 vaccine, while the control group was immunized with saline solution. The mice received two immunization doses of 20 microliters each, containing approximately 400 viral particles, divided between both nostrils, with a 15‐day interval between doses. After 7 days from the last immunization, the mice were sedated with xylazine/ketamine and euthanized for spleen collection.

#### 5.8.2. Isolation of Splenocytes

The spleen was removed from each mouse and mashed onto a 40 µM sieve. Then, 3 mL of PBS was added, and the volume was brought up to 6 mL by slowly adding 3 mL of Histopaque (10831, Sigma–Aldrich). The tube was centrifuged at 400 × *g* for 30 min at room temperature. At the end of centrifugation, the opaque interface with mononuclear cells was separated and transferred to another tube. The cells were washed three times with PBS at 250 × *g* for 10 min at room temperature. The mononuclear cells were ultimately resuspended in 2 mL of FACS medium, and their concentration was determined using a Neubauer chamber.

#### 5.8.3. Cell Staining and Flow Cytometry

The single‐cell suspensions of splenocytes were counted, diluted in 0.4% trypan blue solution, and 10^6^ viable cells per well were seeded in a 96‐well U‐bottom plate. Cell surface antigens were stained with the following monoclonal antibodies. APC‐eFluor 780‐conjugated anti‐CD3 mAb (clone 145‐2C11, 47‐0031‐82), Alexa Fluor 488‐conjugated anti‐CD4 mAb (clone GK1.5, 53‐0041‐82), Super Bright 436‐conjugated anti‐CD8 mAb (clone 53‐6.7, 62‐0081‐82), and PE‐conjugated anti‐CD119 mAb (clone 2E2, 12‐1191‐82), or the respective isotype‐matched control antibodies (47‐4888‐80, 53‐4031‐80, 62‐4321‐80, and 12‐4888‐81, respectively). After a 30‐min incubation, cells were washed with PBS containing 1% fetal bovine serum, resuspended in fixation buffer, and incubated for 20 min. Cells were then permeabilized and resuspended in FACS Buffer with PerCP‐Cy 5.5‐conjugated anti‐TBET mAb or an isotype control, followed by a 20‐min incubation. After washing, cells were transferred to FACS tubes and analyzed using a BD FACSCelesta flow cytometer, with data processed in FlowJo v. 9.9.6.

### 5.9. Syrian Hamster Challenge With Wuhan and Delta Variants and Lung Histopathological Testing

Male Syrian hamsters, aged 6–7 weeks, the animals were individually acclimatized in polypropylene cages with access to water and ad libitum feed, maintained at 25 ± 2°C under a 12‐h light–dark cycle. For the immunization assay, the animals were randomly divided into five groups (Table 2). After 7 days from the last immunization, the animals were challenged with 2 × 10^3^ viral particles of the Wuhan and Delta strain of SARS‐CoV‐2.

**Table 2:** Syrian Hamster experimental groups and immunization protocol.

Groups		Immunization protocol			Viral challenge
		Immunogen	Adjuvant/volume	Immunization timing	
Challenged	Intranasal IBV (*n* = 10 hamster)	IBV‐H120 strain (400 viral particles)	None/25 µL (12.5 µL per nostril)	First immunization (Day 0) Booster dose (Day 14^th^)	Intranasal administration of 2 × 10^3^ SARS‐CoV‐2 viral particles (21^st^ day)
	Subcutaneous IBV (*n* = 10 hamster)	IBV‐H120 strain (400 viral particles)	Aluminum hydroxide (0.05 M)/100 µL		
	Placebo (*n* = 10 hamster)	PBS (intranasal)	None/25 µL (12.5 µL per nostril)		
Control	Intranasal IBV (*n* = 10 hamster)	IBV‐H120 strain (400 viral particles) intranasal	None/25 µL (12.5 µL per nostril)		None
	Healthy (*n* = 10 hamster)	None	None	Not applicable	None

All groups were monitored for 96 h postchallenge. The animals were then euthanized with 200 mg/kg ketamine and 10 mg/kg xylazine (i.p.). The right lung lower lobe was collected, fixed in 10% neutral‐buffered formalin, dehydrated, and paraffin‐embedded. Tissue sections (5 μm) were stained with hematoxylin–eosin and examined by light microscopy (40x or 200x magnification). Histopathological injury was blindly scored as: 0 = No injury; +1 = infiltration with/without peribronchial edema; +2 = peribronchial/intra‐alveolar infiltration, parenchymal edema, septal thickening; +3 = +2 with alveolar consolidation [60]. The Delta strain of SARS‐CoV‐2 was subsequently used to repeat the complete immunization procedure and analysis as outlined in Section 2.5.

### 5.10. Statistical Analysis

Correlation analysis (Pearson’s *r*) and linear regression were used to relate each sample’s absorbance to the degree of neutralization obtained in the PRNT assay. Antibody titer analysis was performed by ANOVA followed by *t*‐test and Tukey test. For histopathology score analysis, scores were expressed as median and range. Data were analyzed using the Kruskal–Wallis test followed by Dunn’s test. For the analysis of flow cytometry data, the nonparametric Mann–Whitney test was used for all comparisons. The intensity and frequency of the samples in flow cytometry were compared using the MANOVA test (Pillai test and Wilks lambda test). All statistical analyses were performed using GraphPad Prism (v.8.0), with a significance level of *p*  < 0.05 or Jasp (v. 0.95.1) with a significance level of *p*  < 0.05.

**Nomenclature**
AdV: Adenovirus
ANOVA: Analysis of variance
BIRD: Binding site, interaction energy, and residue domain
CD4^+^ T cells: Cluster of differentiation 4 positive T cells
COVID‐19: Coronavirus disease 2019
DFT: Density functional theory
EDTA: Ethylenediaminetetraacetic acid
ELISA: Enzyme‐linked immunosorbent assay
FBS: Fetal bovine serum
GGA: Generalized gradient approximation
H&E: Hematoxylin and eosin
IBV: Infectious bronchitis virus
IFN‐γ: Interferon‐gamma
IgA: Immunoglobulin A
IgG: Immunoglobulin G
IgM: Immunoglobulin M
MERS‐CoV: Middle East respiratory syndrome coronavirus
MHC: Major histocompatibility complex
MFCC: Mel‐frequency cepstral coefficients
OIE: World Oorganization for Animal Health
PBE: Perdew, Burke, and Ernzerhof
PBS: Phosphate‐buffered saline
PCR: Polymerase chain reaction
PFU: Plaque‐forming unit
PRNT: Plaque reduction neutralization test
PRNT50: Plaque reduction neutralization test 50
RBD: Receptor‐binding domain
RMSD: Root‐mean‐square deviation
RT‐qPCR: Reverse transcription quantitative polymerase chain reaction
SARS‐CoV‐2: Severe acute respiratory syndrome coronavirus 2
SD: Spike domain
TCID50: Tissue culture infective dose 50
TNF‐α: Tumor necrosis factor‐alpha
Th1: T helper 1
Th2: T helper 2.

## Author Contributions

**Ney de Carvalho Almeida:** conceptualization, formal analysis, investigation, writing – original draft, writing – review and editing. **Mauricio Fraga van Tilburg:** methodology, validation, formal analysis, investigation, writing – original draft, writing – review and editing. **Bruno Bezerra da Silva, João Xavier da Silva Neto, and Luiz Francisco Wemmenson Gonçalves Moura:** investigation, visualization, writing – review and editing. **Natália do Vale Canabrava, Diane Isabelle Magno Cavalcante, Deysi Viviana Tenazoa Wong, Roberto César Pereira Lima, Daniel Pascoalino Pinheiro, and Danúbio Andrade Bezerra Farias:** investigation, validation. **Dayane Alves Costa, and Gilvan Pessoa Furtado:** investigation. **Pablo Abreu de Morais, Valder Nogueira Freire, Valdir Ferreira de Paula, Francisco Franciné Maia, Diego Veras Wilke:** formal analysis, investigation. **Eridan Orlando Pereira Tramontina Florean:** validation, writing – review and editing. **Maria Izabel Florindo Guedes:** conceptualization, methodology, validation, formal analysis, investigation, writing – original draft, writing – review and editing, project administration, funding acquisition.

## Funding

This study was supported by the Fundação Cearense de Apoio ao Desenvolvimento Científico e Tecnológico (FUNCAP) (Process Number 09565434/2022). The Brazilian Agencies: Coordenação de Aperfeiçoamento de Pessoal de Nível Superior and Conselho Nacional de Desenvolvimento Científico e Tecnológico (CNPq) also supported the execution of this project by granting scholarships.

## Conflicts of Interest

The authors declare no conflicts of interest.

## Supporting Information

Additional supporting information can be found online in the Supporting Information section.

## Supporting information


**Supporting Information** Figure S1: Correlation of serum antibody levels and plaque reduction neutralization tests (PRNT) in farm workers. (A) IgM, (B) IgG, and (C) IgA anti‐IBV in SARS‐CoV‐2 neutralization. (D) IgM, (E) IgG, and (F) IgA anti‐SARS‐CoV‐2 in SARS‐CoV‐2 neutralization. Figure S2: Enzyme‐linked immunosorbent assay (ELISA) with serum samples from farm workers, showing the detection of polyclonal antibodies (IgG, IgA, and IgM) that specifically recognized purified SARS‐CoV‐2 and commercial IBV‐H120. Serum samples were analyzed at a final dilution of 1:100: (A) Plate sensitized with purified SARS‐CoV‐2. (B) Plate sensitized with IBV‐H120 (commercial). (C) Neutralization capacity of serum from poultry farm workers (P1–P10) against SARS‐CoV‐2. Each quadrant represents results obtained in duplicate. P, poultry farm worker; C+, positive control; C–, negative control. Figure S3: Flow cytometry analysis of lymphocytes from mice immunized with IBV‐H120. Analysis strategies. (A) Time. (B) Lymphocyte population of the sample. (C) Analyzed singlets. (D) CD3^+^. (E) CD4^+^. (F) CD4^+^/Tbet^+^. (G) CD8^+^. (H) CD8^+^/CD119^+^. (I) Vaccinated CD119^+^. (J) Control CD119^+^. SSC‐A, side scatter area. FSC‐A, foward scatter area. FSC‐H, forward scatter height. Figure S4: Intranasal IBV‐H120 vaccination attenuates the SARS‐Cov‐2‐induced (Wuhan) histopathological injury in the lungs. (A) Healthy; (B) subcutaneous IBV‐H120; (C) placebo; (D) intranasal IBV‐H120. The groups showed in Subfigures (B–D) were challenged by intranasal administration of 2 × 10^3^ SARS‐CoV‐2 (Wuhan strain) viral particles. Lung sections (5 μm) were obtained for hematoxylin–eosin staining (H&E) and analysis under light microscopy (magnification 40x). The black arrow indicates peribronchial inflammatory infiltration; the yellow arrow denotes consolidation of the alveolar airspaces; the green arrow points to reactive epithelial degeneration. Scale bar = 250 μm. Figure S5: Intranasal IBV‐H120 vaccination attenuates the SARS‐CoV‐2‐induced (Delta variant) histopathological injury in the lungs. (A) Healthy; (B) intranasal IBV‐H120; (C) placebo; (D) intranasal IBV‐H120. The groups showed in Subfigures (C, D) were challenged by intranasal administration of 2 × 10^3^ SARS‐CoV‐2 (Delta strain) viral particles. Lung sections (5 μm) were obtained for hematoxylin–eosin staining (H&E) and analysis under light microscopy (magnification 40x). The black arrow indicates peribronchial inflammatory infiltration; the yellow arrow denotes consolidation of the alveolar airspaces; the green arrow points out reactive epithelial degeneration.

## Data Availability

Due to the volume of data generated, some results that support the main findings of this study have been provided as supporting information. These materials offer complementary information that reinforces the conclusions presented in the manuscript.
