# Supplementary material for: Attenuated Infectious Bronchitis Virus (IBV) Vaccination Induces Protective Humoral and Cellular Immunity Against SARS‐CoV‐2 in Mammals
Source: J Immunol Res. 2026 Mar 29;2026:9309800. doi: 10.1155/jimr/9309800 (PMC13140183; doi:10.1155/jimr/9309800)
Supplement: Supplementary file 1 — Supporting Information Figure S1: Correlation of serum antibody levels and plaque reduction neutralization tests (PRNT) in farm workers. (A) IgM, (B) IgG, and (C) IgA anti‐IBV in SARS‐CoV‐2 neutralization. (D) IgM, (E) IgG, and (F) IgA anti‐SARS‐CoV‐2 in SARS‐CoV‐2 neutralization. Figure S2: Enzyme‐linked immunosorbent assay (ELISA) with serum samples from farm workers, showing the detection of polyclonal antibodies (IgG, IgA, and IgM) that specifically recognized purified SARS‐CoV‐2 and commercial IBV‐H120. Serum samples were analyzed at a final dilution of 1:100: (A) Plate sensitized with purified SARS‐CoV‐2. (B) Plate sensitized with IBV‐H120 (commercial). (C) Neutralization capacity of serum from poultry farm workers (P1–P10) against SARS‐CoV‐2. Each quadrant represents results obtained in duplicate. P, poultry farm worker; C+, positive control; C–, negative control. Figure S3: Flow cytometry analysis of lymphocytes from mice immunized with IBV‐H120. Analysis strategies. (A) Time. (B) Lymphocyte population of the sample. (C) Analyzed singlets. (D) CD3+. (E) CD4+. (F) CD4+/Tbet+. (G) CD8+. (H) CD8+/CD119+. (I) Vaccinated CD119+. (J) Control CD119+. SSC‐A, side scatter area. FSC‐A, foward scatter area. FSC‐H, forward scatter height. Figure S4: Intranasal IBV‐H120 vaccination attenuates the SARS‐Cov‐2‐induced (Wuhan) histopathological injury in the lungs. (A) Healthy; (B) subcutaneous IBV‐H120; (C) placebo; (D) intranasal IBV‐H120. The groups showed in Subfigures (B–D) were challenged by intranasal administration of 2 × 10^3^ SARS‐CoV‐2 (Wuhan strain) viral particles. Lung sections (5 μm) were obtained for hematoxylin–eosin staining (H&E) and analysis under light microscopy (magnification 40x). The black arrow indicates peribronchial inflammatory infiltration; the yellow arrow denotes consolidation of the alveolar airspaces; the green arrow points to reactive epithelial degeneration. Scale bar = 250 μm. Figure S5: Intranasal IBV‐H120 vaccination attenuates the SARS‐C [file JIMR-2026-9309800-s001.docx]

**Attenuated Infectious Bronchitis Virus (IBV) Vaccination Induces Protective Humoral and Cellular Immunity Against SARS-CoV-2 in Mammals**

**Supplementary metodology**

- ***In Silico* Methods**
- **Target editing**

The protein structures were subjected to editing the missing residues with swissPDBviewer [1], followed by protonation adjustment in the APBS web service [2]. Subsequently, the spike protein was cut using PyMOL software [3] to obtain the region of interest.

The missing residues of the structures were added using SwissPDBviewer [1], and their protonation states were adjusted using the APBS web service [2]. Subsequently, we extracted the region of interest of the spike protein using PyMOL software [3].

- **Molecular Docking**

The IBV spike/P2B-2F6 complex was subjected to refinement by the Patchdock server [4] and docking on the FireDock server [5] with the default parameters, obtaining a molecular docking position cluster classifying the models with (Global Energy; aVdW, rVdW; Waals ACE; aElec,rElec; laElec, lrElec; HB; piS; catpiS; aliph; Transformation). Finally, the protein-antibody binding affinity was evaluated by the PRODIGY server (ΔG) [6].

- **Molecular Dynamics (MD)**

Atomistic simulations with GROMACS v2021 [7] were performed to evaluate the structural changes in the IBV S/P2B-2F6 complex, using the field force CHARMM36. Subsequently, the system was solvated into an orthorhombic periodic box of TIP3P water molecules which was extended 20 Å from any solute atom. In the solvation, 158833 water molecules were added as well as 18 Na^+^ ions to neutralize the system [8]. The electrostatic interaction was computed using the fast Particle-Mesh Ewald approach, while the van der Waals interaction (vdW) was calculated using the cutoff scheme. Initially, the complex was submitted to energy minimization using the steepest descent approach. The temperature balance of the system was set to 300 K through the NVT assembly and with the pressure balance using the Berendsen coupling and compressibility of 4.5 · 10-5 bar in NPT. MD simulations were performed for a time interval of 120 ns and the dynamic stability of the complex was calculated as the root mean square deviation (RMSD).

**References**

1. Guex N, Peitsch MC. SWISS-MODEL and the Swiss-Pdb Viewer: An environment for comparative protein modeling. Electrophoresis. 1997;18(15):2714–23.

2. Jurrus E, Engel D, Star K, Monson K, Brandi J, Felberg LE, et al. Improvements to the APBS biomolecular solvation software suite. Protein Science. 2018 Jan 24;27(1):112–28.

3. Schrödinger L, DeLano W. PyMOL. 2020.

4. Andrusier N, Nussinov R, Wolfson HJ. FireDock: Fast interaction refinement in molecular docking. Proteins: Structure, Function, and Bioinformatics. 2007 Oct 27;69(1):139–59.

5. Mashiach E, Schneidman-Duhovny D, Andrusier N, Nussinov R, Wolfson HJ. FireDock: a web server for fast interaction refinement in molecular docking. Nucleic Acids Research. 2008 May 19;36(Web Server):W229–32.

6. Xue LC, Rodrigues JP, Kastritis PL, Bonvin AM, Vangone A. PRODIGY: a web server for predicting the binding affinity of protein–protein complexes. Bioinformatics. 2016 Dec 1;32(23):3676–8.

7. Abraham MJ, Murtola T, Schulz R, Páll S, Smith JC, Hess B, et al. GROMACS: High performance molecular simulations through multi-level parallelism from laptops to supercomputers. SoftwareX. 2015 Sep;1–2:19–25.

8. Jorgensen WL, Chandrasekhar J, Madura JD, Impey RW, Klein ML. Comparison of simple potential functions for simulating liquid water. The Journal of Chemical Physics. 1983;79(2):926–35.

**Supplementary figures captions:**


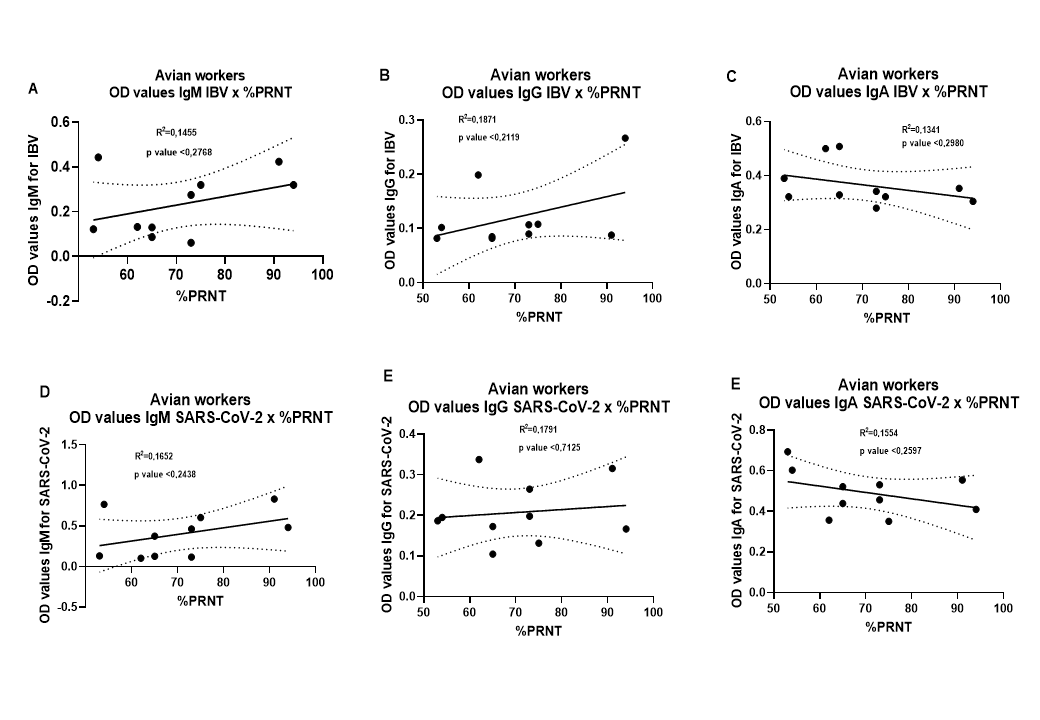


**Figure Supplementary 1:** Correlation of serum antibody levels and plaque reduction neutralization tests (PRNT) in farm workers. (A) IgM, (B) IgG, and (C) IgA anti-IBV in SARS-CoV-2 neutralization. (D) IgM, (E) IgG, and (F) IgA anti-SARS-CoV-2 in SARS-CoV-2 neutralization.


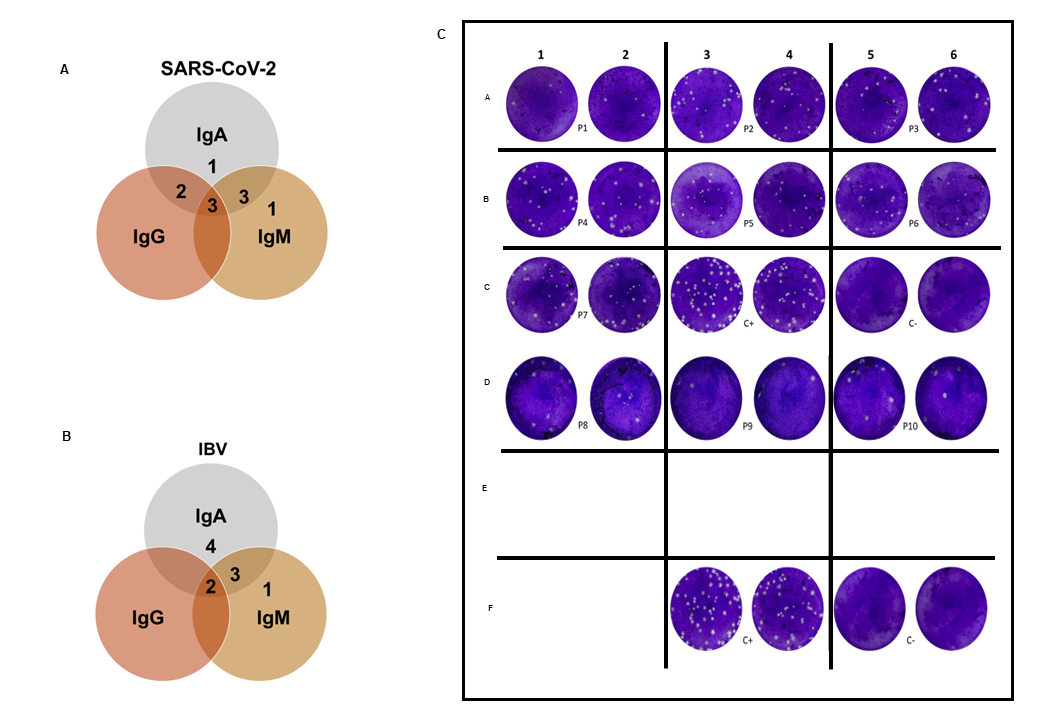


**Figure Supplementary 2:** Enzyme-linked immunosorbent assay (ELISA) with serum samples from farm workers, showing the detection of polyclonal antibodies (IgG, IgA, and IgM) that specifically recognized purified SARS-CoV-2 and commercial IBV-H120. Serum samples were analyzed at a final dilution of 1:100: (A) Plate sensitized with purified SARS-CoV-2. (B) Plate sensitized with IBV-H120 (commercial). (C) Neutralization capacity of serum from poultry farm workers (P1–P10) against SARS-CoV-2. Each quadrant represents results obtained in duplicate. P = poultry farm worker; C+ = positive control; C– = negative control.


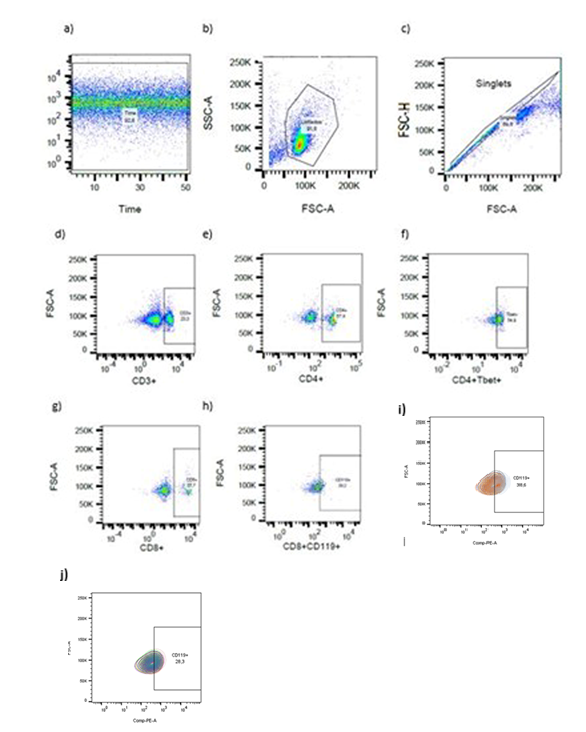


**Figure Supplementary 3:** Flow cytometry analysis of lymphocytes from mice immunized with IBV-H120. Analysis strategies. A) Time. B) Lymphocyte population of the sample. C) Analyzed singlets. D) CD3+. E) CD4+. F) CD4+ / Tbet+. G) CD8+. H) CD8+ / CD119+. I) Vaccinated CD119+ J) Control CD119+. SSC-A= Side scatter area. FSC-A = Foward scatter area. FSC-H= Forward scatter height.


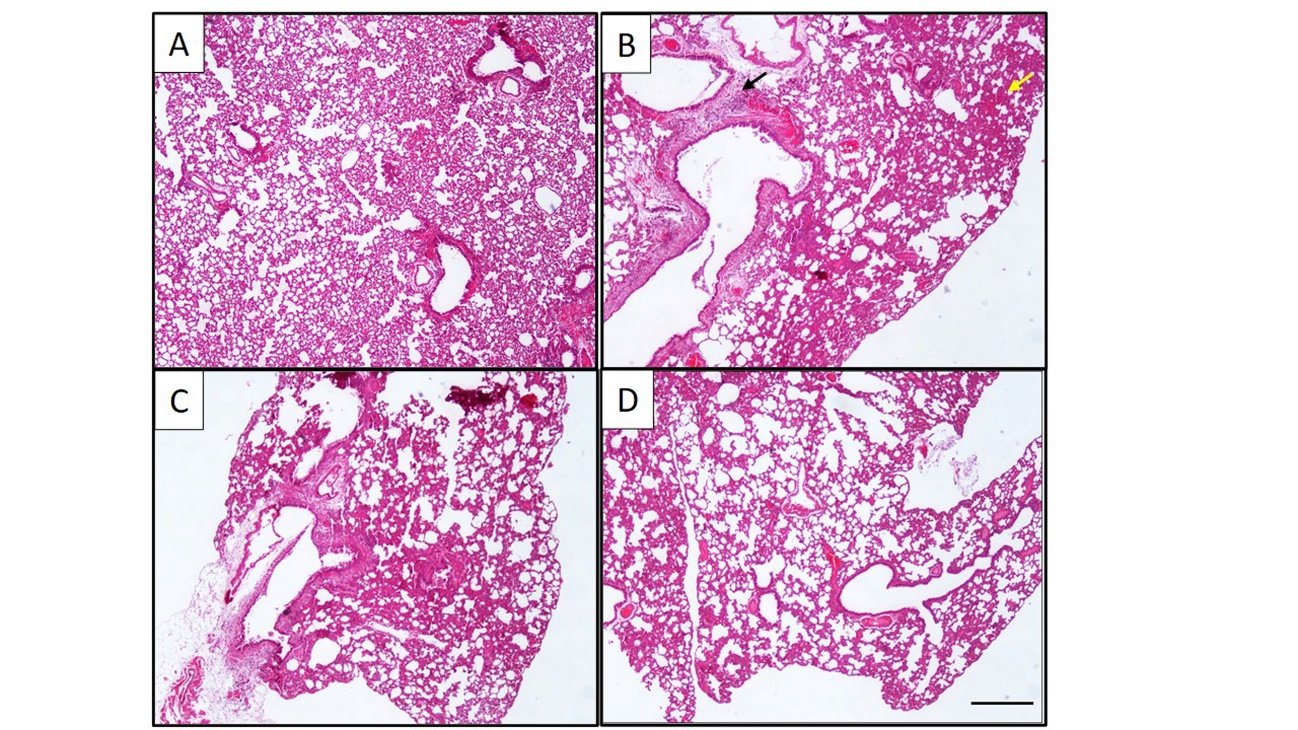


**Figure Supplementary 4:** Intranasal IBV-H120 vaccination attenuates the SARS-Cov-2-induced (Wuhan) histopathological injury in the lungs. A = Healthy; B = Subcutaneous IBV-H120; C = Placebo; D = Intranasal IBV-H120. The groups showed on B, C, and D were challenged by intranasal administration of 2 × 10³ SARS-CoV-2 (Wuhan strain) viral particles. Lung sections (5 μm) were obtained for hematoxylin-eosin staining (H&E) and analysis under light microscopy (Magnification 40x). The black arrow indicates peribronchial inflammatory infiltration; the yellow arrow denotes consolidation of the alveolar airspaces; the green arrow points to reactive epithelial degeneration. Scale bar = 250 μm.


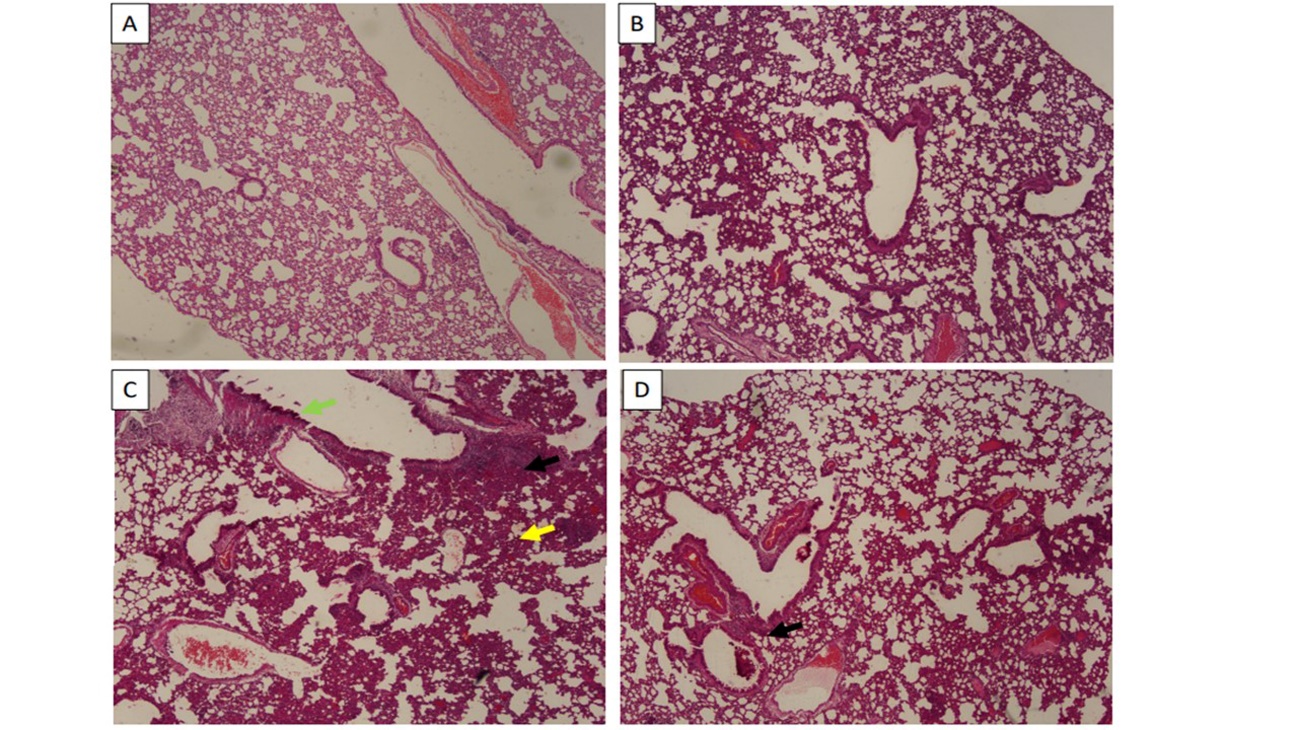


**Figure Supplementary 5:** Intranasal IBV-H120 vaccination attenuates the SARS-CoV-2-induced (Delta variant) histopathological injury in the lungs.A = Healthy; B = Intranasal IBV-H120; C = Placebo; D = Intranasal IBV-H120. The groups showed on C and D were challenged by intranasal administration of 2 × 10³ SARS-CoV-2 (Delta strain) viral particles. Lung sections (5 μm) were obtained for hematoxylin-eosin staining (H&E) and analysis under light microscopy (Magnification 40x). The black arrow indicates peribronchial inflammatory infiltration; the yellow arrow denotes consolidation of the alveolar airspaces; the green arrow points out reactive epithelial degeneration.
